# Identification of cleavage sites and substrate proteins for two mitochondrial intermediate peptidases in *Arabidopsis thaliana*

**DOI:** 10.1093/jxb/erv064

**Published:** 2015-03-01

**Authors:** Chris Carrie, A. Saskia Venne, René P. Zahedi, Jürgen Soll

**Affiliations:** ^1^Department of Biology I, Botany, Ludwig-Maximilians Universität München, Großhaderner Strasse 2–4, D-82152 Planegg-Martinsried, Germany; ^2^Leibniz-Institut für Analytische Wissenschaften - ISAS - e.V., Otto-Hahn-Str. 11, D-44139 Dortmund, Germany; ^3^Munich Centre for Integrated Protein Science, CiPSM, Ludwig-Maximilians Universität München, Feodor-Lynen-Strasse 25, D-81377 Munich, Germany

**Keywords:** ChaFRADIC method, Icp55, mitochondrial processing peptidase (MPP), Oct1, –10R motif.

## Abstract

Most mitochondrial proteins contain an N-terminal targeting signal that is removed by specific proteases following import. In plant mitochondria, only mitochondrial processing peptidase (MPP) has been characterized to date. Therefore, we sought to determine the substrates and cleavage sites of the *Arabidopsis thaliana* homologues to the yeast Icp55 and Oct1 proteins, using the newly developed ChaFRADIC method for N-terminal protein sequencing. We identified 88 and seven putative substrates for *Arabidopsis* ICP55 and OCT1, respectively. It was determined that the *Arabidopsis* ICP55 contains an almost identical cleavage site to that of Icp55 from yeast. However, it can also remove a far greater range of amino acids. The OCT1 substrates from *Arabidopsis* displayed no consensus cleavage motif, and do not contain the classical –10R motif identified in other eukaryotes. *Arabidopsis* OCT1 can also cleave presequences independently, without the prior cleavage of MPP. It was concluded that while both OCT1 and ICP55 were probably acquired early on in the evolution of mitochondria, their substrate profiles and cleavage sites have either remained very similar or diverged completely.

## Highlight

This study has identified two conserved mitochondrial peptidases in plants. Compared to other eukaryotes, AtICP55 has a conserved function and cleavage motif, and AtOCT1 has a completely novel cleavage motif.

## Introduction

Despite mitochondria possessing their own genome, the vast majority of mitochondrial proteins are encoded in the nucleus and are imported post-translationally following synthesis on cytosolic ribosomes. In plants, it has been estimated that somewhere between 1000 and 2000 proteins must be imported into the mitochondrion to ensure proper function (Millar *et al.*, 2005). Most proteins that are destined for mitochondria contain N-terminal targeting signals called presequences, which are cleaved off following import. Presequences are generally positively charged and are able to form amphiphilic α-helices, but do not have any primary amino acid conservation (von Heijne, 1986; von Heijne *et al.*, 1989). The main role of the presequence is to direct mitochondrial proteins to the mitochondria, where they interact with the TOM (‘Translocase of the outer membrane’) complex, and are then passed through the outer membrane to the TIM (‘Translocase of the inner membrane’) complex for passage through the inner membrane into the matrix (Chacinska *et al.*, 2009; Becker *et al.*, 2012). Once inside the matrix, the presequences are cleaved off by the mitochondrial processing peptidase (MPP) (Hawlitschek *et al.*, 1988).

MPP is a metalloendopeptidase which forms a heterodimer consisting of two structurally related α and β subunits (Glaser *et al.*, 1998). While MPP has been found in all mitochondria to date, its intra-organellar location can differ. In yeast and humans, MPP is found soluble in the matrix (Ou *et al.*, 1989), while in plants it is completely integrated into the bc_1_ complex of the respiratory chain (Braun *et al.*, 1992; Glaser and Dessi, 1999; Dessi *et al.*, 2000). Even though MPP is found in different locations in different organisms, its role and cleavage motifs have remained very similar (Teixeira and Glaser, 2013). Early reports on MPP processing sites in yeast model systems split them into four different groups. The first two groups make up ~80% of presequences, and contain either an arginine at position –2 (–2R) or –3 (–3R) relative to the cleavage site and have a loosely conserved cleavage motif of RX↓X and RX(F/Y/L)↓(A/S)X, respectively (where ↓ is the cleavage site and X is any amino acid) (Gakh *et al.*, 2002). The third group of presequences contain no conserved arginine (no-R) at the processing site (Zhang *et al.*, 2001), and the final group contain an arginine at the –10 positon (–10R) with the consensus motif RX↓(F/L/I)X_2_(T/S/G)X_4_↓X (Gakh *et al.*, 2002). Studies on plant mitochondria determined that the –2R and –3R motifs were also used in all the species examined (Zhang *et al.*, 2001; Huang *et al.*, 2009). In addition, plant mitochondrial presequences contain no-R motifs with the cleavage motif (F/Y)↓(S/A) (Huang *et al.*, 2009). Interestingly, the –10R motif has not been identified in any plant mitochondrial presequence (Teixeira and Glaser, 2013).

Further work on mitochondrial presequence processing has demonstrated that a number of mitochondrial presequences are not only processed by MPP, but can undergo a second proteolytic event following MPP cleavage (Gakh *et al.*, 2002; Teixeira and Glaser, 2013). Originally, the necessity for these secondary cleavage events was unclear; however, recent work in yeast has demonstrated that these second-processing steps are important for protein stability within the mitochondria (Vogtle *et al.*, 2009; Vogtle *et al.*, 2011). The reason for some secondary processing remains unclear (Vogtle *et al.*, 2011). So far, two proteases have been implicated in secondary processing in mitochondria. The first has been known for over 20 years and is called Oct1 (‘Octapeptidyl aminopeptidase 1’; also known as mitochondrial intermediate peptidase, MIP) and has been shown to cleave an octapeptide from the MPP-generated N-terminus (Isaya *et al.*, 1991; Isaya *et al.*, 1994; Vogtle *et al.*, 2011). Oct1 processing is responsible for the –10R motif in yeast mitochondrial presequences. While homologues of Oct1 have been identified in plants, as yet there are no functional data on their role.

The next secondary processing peptidase to be identified was Icp55 (‘Intermediate cleaving peptidase of 55 kDa’) (Vogtle *et al.*, 2009). Icp55 has been demonstrated to cleave one amino acid from the MPP-generated intermediate N-terminus. In yeast, Icp55 is specific for the substrates containing the consensus motif (Y/L/F)↓(S/A) (Vogtle *et al.*, 2009). The identification of Icp55 resolved the issue of the –2R vs –3R MPP recognition motifs in yeast, as it demonstrated that the –3R motif is actually a –2R followed by the removal of a single amino acid by Icp55. The interesting feature linking Icp55 and Oct1 is that the secondary processing converts a primary destabilizing amino acid at the N-terminus generated by MPP cleavage into a stabilizing one, in accordance with the N-end rule for protein stability (Bachmair *et al.*, 1986; Vogtle *et al.*, 2009; Varshavsky, 2011; Vogtle *et al.*, 2011). It has been further demonstrated that inactivation of either Icp55 or Oct1 leads to the decreased half-lives of their substrates (Vogtle *et al.*, 2009; Vogtle *et al.*, 2011).

Since it has been established that Icp55 and Oct1 are important for protein stability within mitochondria, and since both proteins have homologues in plants, this study set out to determine the substrates and cleavage sites of the AtICP55 and AtOCT1 proteins in *Arabidopsis thaliana*. T-DNA knockout lines were isolated and mitochondria were analysed using the ChaFRADIC (charge-based fractional diagonal chromatography) technique to identify N-terminal peptides from the knockout lines and wild type. ChaFRADIC has been previously used to determine a number of Icp55 substrates in yeast mitochondria (Venne *et al.*, 2013). In this study we identified 88 potential substrates for AtICP55 and seven for AtOCT1. In contrast to AtICP55, AtOCT1-processing specificity showed little conservation with that from yeast. Therefore, we have identified two conserved intermediate peptidases in *Arabidopsis*, one (AtICP55) with a conserved cleavage site between yeast and plants and a second (AtOCT1) which appears to have a similar role to that from yeast but a completely different cleavage site motif.

## Materials and methods

### cDNA clones and constructs

The chromosomal loci for potential AtICP55 (At1g09300) and AtOCT1 (At5g51540) have been previously identified (Kwasniak *et al.*, 2012). The full-length coding sequences of AtICP55, AtOCT1, NADH B13 (At5g52840), PMH2 (At3g22330), PRORP1 (At2g32230), and RISP1 (At5g13430) were cloned into vector pDest14 (Invitrogen) using gateway (Invitrogen) cloning techniques for *in vitro* transcription and translation. The cDNA clones for AOX (X68702) and Tim23 (At1g72750) have been described previously (Whelan *et al.*, 1995b; Murcha *et al.*, 2003). For GFP analyses, the full-length coding sequence and the first 100 amino acids of AtICP55 and AtOCT1 were cloned upstream of GFP under the control of a constitutive promoter in the vector pK7FWG2 (Karimi *et al.*, 2002).

### Subcellular localization of GFP

The *Agrobacterium tumefaciens* strain AGL1 transformed with the appropriate GFP construct was used to infiltrate 4- to 6-week-old *Nicotiana benthamiana* leaves as described previously (Schweiger *et al.*, 2012). Protoplasts were prepared as outlined in Koop *et al.* (1996) except that cell walls were digested for 90min at 40rpm in 1% cellulase R10 and 0.3% macerase R10 after vacuum infiltration. Mitotracker (Life Technologies) was added to protoplast suspensions to a final concentration of 500nM. Fluorescence was observed with a confocal laser scanning microscope at 20°C (Leica, Type TCS SP5).

### T-DNA insertion lines

The following T-DNA insertion lines were obtained from the Nottingham Arabidopsis Stock Centre (NASC) and genotyped by PCR to confirm homozygosity for the T-DNA insert: *Δicp55* - Sail_672_D05, *Δoct1-1* - SALK_018660C, and *Δoct1-2* - SALK_057646C.

### Mitochondrial isolation

Mitochondria for *in vitro* import assays and the ChaFRADIC analysis were harvested from 20g (approximate fresh weight) of 14-day-old *Arabidopsis thaliana* seedlings grown in liquid culture as previously described (Lister *et al.*, 2007). The only change made was for the ChaFRADIC analysis, whereby a second Percoll gradient was performed to improve the purity of mitochondria. Typically 2–3mg of mitochondrial protein was obtained from each preparation.

### ChaFRADIC analyses

The ChaFRADIC-based enrichment of N-terminal peptides was conducted as described previously (Venne *et al.*, 2013). Briefly, each sample was lysed (1% SDS, 50mM HEPES, pH 7.8) on ice followed by heating to 85°C for 5min. After ethanol precipitation, protein pellets were resolubilized (6M GuHCl, 50mM HEPES, pH 7.8), followed by determination of protein concentration using bicinchoninic acid (BCA) assay (Thermo Scientific). Cysteines were reduced by addition of 10mM dithiothreitol for 30min at 56°C, and free thiols carbamidomethylated by adding 20mM iodoacetaminde for 30min at room temperature in the dark. Afterwards, aliquots corresponding to 80 µg of protein per condition were used. Free N-termini and lysine residues were blocked by dimethylation using light (wild type, 28Da) and medium (mutant, 34Da) label. Next, wild-type and mutant samples were pooled in a 1:1 ratio and subjected to ethanol precipitation. After resolubilizing the pellet (2M GuHCl, 50mM Na_2_HPO_4_, pH 7.8) the sample was diluted 10-fold (50mM NH_4_HCO_3_, 5% ACN, 1mM CaCl_2_). Tryptic digestion was performed for 15h at 37°C using a 1:25 ratio (trypsin:protein, w/w). Afterwards, digestion efficiency was controlled as described previously (Burkhart *et al.*, 2012). Generated peptides were desalted using C18 solid-phase tips (SPEC C18 AR, 4mg bed, Agilent Technologies) according to the manufacturer’s instructions and peptides were dried under vacuum, followed by resolubilization in SCX buffer A (10mM KH_2_PO_4_, 20% ACN, pH 2.7).

Collected fractions were dried under vacuum and purified for LC-MS measurement using C18 Supel pipette tips (Sigma-Aldrich) according to the manufacturer’s instructions. Dried C18 eluates were resuspended in 0.1% TFA and analysed by nano-LC-MS/MS using a Q-Exactive mass spectrometer (Thermo Scientific) online-coupled to a nano-RSLC system (both Thermo Scientific). Samples were analysed in data-dependent acquisition mode acquiring full MS scan at R = 70 000, followed by MS/MS of the 15 most abundant ions (Top15) at R = 17 500. For the full scan, target value and maximum injection time were set to 3×10^6^ ions and 120ms, respectively. For MS/MS scans these values were set to 2×10^5^ ions and 250ms. Peptides were fragmented with a normalized collision energy of 30 and for fractions +3, +4, and +5, 10% NH_4_OH solution was placed in front of the ion source for charge state reduction, as described by Thingholm *et al.* (2010)

Raw data generated was searched against an *Arabidopsis* Uniprot database (July 2012; 11 340 target sequences) using Proteome Discoverer version 1.3 (Thermo Scientific) with Mascot 2.4 (Matrix Science) and the precursor ion quantifier and peptide validator nodes. To enable the quantification of endogenously acetylated as well as N-terminally dimethylated peptides we used a two-step strategy with semi ArgC enzyme specificity and a maximum of two missed cleavage sites: First, dimethylation light (+28.0313Da) and dimethylation medium (+34.0689Da) of Lys and N-termini were allowed as variable modifications and carbamidomethylation of Cys (+ 57.0214Da) was allowed as a fixed modification. Second, N-terminal acetylation (+ 42.0105Da) and dimethylation (light/medium) of Lys were allowed as variable modifications and carbamidomethylation of Cys was allowed as a fixed modification. Mass tolerance was set to 10 ppm (parts per million) for MS and 0.02Da for MS/MS. For quantification, an elution time window of up to 1min was allowed to compensate for deuterium-induced retention time shifts of the medium-labelled peptides. Peptide spectrum matches (PSMs) were filtered for ‘search engine rank 1’ and ‘high confidence’, corresponding to a false discovery rate <1%. Only peptides with blocked N-termini (dimethylated/endogenously acetylated) that were consistently labelled and showing at least a 3-fold change between wild-type and mutant samples were considered as differentially expressed.

### Data analysis

After peptide identification, uniprot IDs were converted into *Arabidopsis* accession numbers using the uniprot website (http://www.uniprot.org). *Arabidopsis* accession numbers were then used to search the SUBA database to first determine the subcellular location and description of protein function (Tanz *et al.*, 2013). Proteins were selected which had a SUBA con score of ‘mitochondrial’ (Hooper *et al.*, 2014). Next, peptides were discarded if they were identified after the 120th amino acid of the full-length peptide. This cut off was applied to discard internal peptides that were probably generated from degradation. Sequence logos were made in the iceLogo program (Colaert *et al.*, 2009). TargetP was used to predict organelle cleavage sites (Emanuelsson *et al.*, 2007). Presequence net charge was calculated for a pH of 7 using the Protein Calculator v3.4 program (http://protcalc.sourceforge.net/). Amino acid frequencies were determined using the ProtParam tool on the expasy website (http://web.expasy.org/protparam/).

### *In vitro* import studies

[^35^S]Met-labelled precursor proteins were synthesized using the Flexi Rabbit Reticulocyte Lysate (Promega) as previously outlined (Chang *et al.*, 2014). The use of equivalent quantities of mitochondria from different genotypes in import reactions was ensured by triplicate measurement of protein concentration using the Lowry method. Normal and time course import experiments into intact mitochondria isolated from different genotypes were performed as described previously (Whelan *et al.*, 1995a; Lister *et al.*, 2007). *In vitro* imports analysed by BN-PAGE gels were performed as outlined in Carrie *et al.* (2010). All *in vitro* imports were obtained using autoradiography and images were scanned using a Typhoon scanner (GE Healthcare).

## Results

### Identification and subcellular localization of AtICP55 and AtOCT1

In previous work, the *Arabidopsis* homologues of the yeast Icp55 and Oct1 proteins were identified as the gene accession numbers At1g09300 (AtICP55) and At5g51540 (AtOCT1) (Kwasniak *et al.*, 2012). We repeated BLAST searches of the *Arabidopsis* genome with the protein sequences of ScIcp55 and ScOct1 and confirmed the same genes as the closest homologues to the yeast proteins (Altschul *et al.*, 1990). However, when looking at the locus At1g09300 for AtICP55, we noticed that the TAIR database predicts two different transcripts for Atlcp55 (At1g09300.1, AtICP55.1; and At1g09300.2, AtICP55.2) which differ at the N-terminus. Protein alignments and identity and similarity percentages suggest that the yeast and *Arabidopsis* proteins are closely related (Supplementary Table S1). Next, we queried the SUBA (Tanz *et al.*, 2013) database to see if any localization data was known for AtICP55.1, AtICP55.2, and AtOCT1. The only experimental data available showed that the AtOCT1 protein had been identified in proteomic studies to localize to plastids (Kleffmann *et al.*, 2004; Peltier *et al.*, 2004) and the plasma membrane (Li *et al.*, 2012a). Further investigation of the SUBA database results, especially the predicted locations, shows that AtICP55.1 and AtOCT1 are predicted by several programs to be mitochondrial and AtICP55.2 shows a combination of both mitochondrial and nuclear predictions. The nuclear prediction for AtICP55.2 is interesting as the ScIcp55 has also been suggested to target the nucleus as well as the mitochondria (Naamati *et al.*, 2009).

**Fig. 1.**
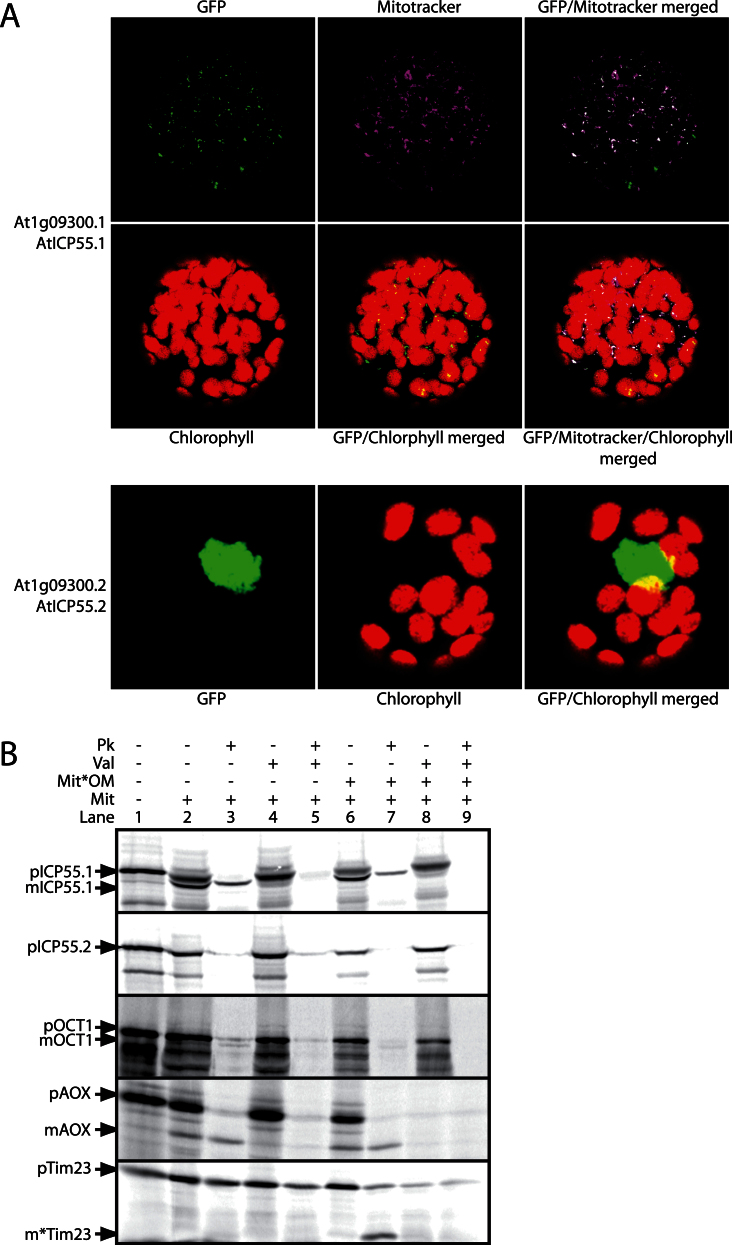
Confirmation of the mitochondrial localization of the AtICP55 and AtOCT1 homologues. (A) Protoplasts of transformed tobacco leaves with the GFP constructs merged to the first 100 amino acids of AtICP55.1 and AtICP55.2 were monitored by confocal laser scanning microscopy. Also shown are mitotracker false-coloured blue for mitochondrial localization and the chlorophyll autofluorescence (red) indicating the location of the chloroplasts. (B) *In vitro import* of radiolabelled AtICP55.1, AtICP55.2, and AtOCT1 into isolated mitochondria. Along with the control proteins AOX and Tim23. lanes contained: 1, precursor protein alone; 2, precursor protein incubated with mitochondria under conditions that support import into mitochondria; 3, as lane 2 but with proteinase K added after incubation of the precursor with mitochondria; 4 and 5, as lanes 2 and 3 but with valinomycin added to the import assay before the addition of precursor protein; lanes 6–9, as lanes 2–5 except that the mitochondrial outer membrane was ruptured after the incubation period with precursor protein, but before the addition of proteinase K. Mit, mitochondria; Mit*OM, mitochondria with the outer membrane ruptured; Pk, proteinase K; Val, valinomycin; p, precursor protein band; m, mature protein band; m*, inner membrane-protected fragment of Tim23.

In order to determine the subcellular targeting of AtICP55.1, AtICP55.2, and Oct1, we performed both *in vivo* GFP tagging experiments and *in vitro* import assays into isolated *Arabidopsis* mitochondria. We fused the full-length coding sequences along with just the first 100 amino acids of all three proteins to the N-terminus of GFP. Expression constructs were then transformed into tobacco and monitored by laser scanning confocal microscopy. We never observed any GFP fluorescence for all the full-length constructs and all constructs of AtOCT1 (data not shown). However, for the constructs encoding the first 100 amino acids of AtICP55.1 and AtICP55.2, we observed good expression of GFP (Fig. 1A). It could be observed that when AtICP55.1 was fused to GFP, it was targeted to mitochondria, which was verified by the overlap of the GFP signal with mitotracker (Fig. 1A). This confirms the prediction that AtICP55.1 is a mitochondrial protein. Interestingly, the GFP signal for AtICP55.2 was observed to be solely in the nucleus, as evidenced by the GFP only fluorescing in one distinct large organelle in the middle of the cell (Fig. 1A). This suggests that the dual localization of Icp55-like proteins between the nucleus and mitochondria is conserved between yeast and *Arabidopsis* (Naamati *et al.*, 2009). While the mode of dual targeting in yeast is unknown, in *Arabidopsis* it is carried out by alternative splicing to create two different transcripts, one encoding the mitochondrial form and the other encoding the nuclear version.

To confirm the results obtained by GFP and to determine whether the AtOCT1 protein is imported into mitochondria, we next performed *in vitro* import assays with isolated *Arabidopsis* mitochondria. Both AtICP55.1 and AtOCT1 were imported into processed protease-protected locations; however, the import of AtOCT1 was very weak as the intensity of the imported band was low (Fig. 1B, lanes 1–5). Both proteins are located either in the matrix or in the matrix side of the inner membrane, as both processed products remain protease protected even after rupture of the outer membrane (Fig. 1B, lanes 6–9). This confirms the observations made using GFP assays and demonstrates that the AtOCT1 protein can also be imported into mitochondria. No import of AtICP55.2 into mitochondria was observed, as no protease-protected band was observed (Fig. 1B), which also confirms the GFP result as not showing a mitochondrial protein, but rather a protein targeted to the nucleus. We also tested whether AtICP55 or AtOCT1 could be imported into isolated chloroplasts; no import was observed (data not shown). Import of the *Glycine max* AOX and *Arabidopsis* Tim23 was used to verify that import was membrane-potential dependent (Fig. 1B, lanes 1–5), and that the outer membrane was successfully ruptured but that the inner membrane was still intact, as demonstrated by the presence of a characteristic membrane-protected fragment of Tim23 and also the presence of AOX upon the addition of a protease when the outer membrane was ruptured (Fig. 1B, lanes 6–9).

### Role of AtICP55.1 and AtOCT1 *in vivo*

After confirming that both AtICP55.1 and AtOCT1 proteins were located in mitochondria, we next wanted to determine their roles in *Arabidopsis* mitochondria. We obtained T-DNA insertion lines: one line for AtICP55 and two independent lines for AtOCT1. Using PCR, these lines were screened for homozygosity and seeds were bulked. T-DNA insertion sites were confirmed by DNA sequencing (Supplementary Figure S1). To confirm that the PCR-screened lines were true knockouts, we amplified the full open-reading frames of both AtICP55 and AtOCT1 from cDNA obtained from RNA isolated from each line. For both AtOCT1 and AtICP55, only a full length transcript was only obtained in the wild-type samples (Supplementary Figure S1). Previous work on yeast mitochondria demonstrated that *ΔScOct1* mitochondria were respiration deficient; *ΔScIcp55* mitochondria showed no significant difference in growth, but displayed a reduced mitochondrial oxygen consumption when grown on glucose as a carbon source (Mossmann *et al.*, 2012; Stames and O’Toole, 2013). In contrast, neither *Δicp55* nor *Δoct1* lines showed any significant difference of phenotype compared to the wild type when grown under a number of conditions (data not shown). However, to determine the roles AtICP55 and AtOCT1 have in the processing of mitochondrial targeting signals, we employed the newly developed ChaFRADIC method to sequence N-terminal peptides from mitochondria isolated from *Arabidopsis*, Col-0 (wild type), and the three T-DNA insertion lines *Δicp55, Δoct1-1*, and *Δoct1-2.*

Mitochondria were isolated from 14-day-old seedlings as described in Lister *et al.* (2007), and 80 µg of mitochondrial protein was used in the ChaFRADIC analysis as described in Venne *et al.* (2013). In order to gain information about the difference in abundance between the wild-type and the T-DNA insertion lines, the wild-type samples were labelled using heavy (mutant lines) and light (wild type) dimethyl labelling before mixing in a 1:1 ratio prior to proteolytic digestion. After N-terminal enrichment, peptides were sequenced by LC-MS analysis.

To identify N-terminal peptides from the data, three criteria were used. First, peptides had to come from mitochondrial proteins that were determined using the SUBA database (Tanz *et al.*, 2013). Only peptides coming from proteins with a SUBAcon score of ‘mitochondrial’ were selected. SUBAcon is a consensus algorithm for identifying subcellular localization using both experimental data and predicted locations (Hooper *et al.*, 2014). Second, peptides that started after the 120th amino acid were discarded as they are unlikely to be true N-terminal peptides. Third, when still left with multiple peptides for proteins, the most N-terminal peptide was selected as the true N-terminus of the protein. Exceptions were made in those cases where abundance differed between the wild type and the mutants. This left, in the wild type:*Δicp55* sample, 248 peptides; in the wild type:*Δoct1-1* sample, 173 peptides; and in the wild type:*Δoct1-2* sample, 194 peptides (Supplementary Table S2).

To verify that we had little or no degradation in the samples, we first analysed proteins that had no identified presequence. In other words, we extracted the proteins whose most N-terminal identified peptide started either with the first methionine or the second amino acid (Supplementary Table S3). This produced a list of 35 proteins with no presequence, 10 of which had also been identified in an earlier study looking at mitochondrial presequences (Huang *et al.*, 2009). In fact, all 10 proteins had identical peptides sequenced in this and the earlier study. The other 25 new N-terminal sequences were mostly from known outer membrane, intermembrane space, or inner membrane proteins. At least two (ATP epsilon, At1g51650; and Carbonic anhydrase 3, At5g66510) of these newly identified proteins have also been shown not to contain cleavable presequences in *in vitro* import assays using isolated mitochondria in previous studies (Li *et al.*, 2012*b*; Li *et al.*, 2013). By analysing proteins which lack a presequence we have shown that we have identified true N-terminal peptides from our samples, indicating little or no degradation. Therefore, the samples were considered reliable for further analysis.

### Identification of AtICP55 substrates

After confirming that the samples contained little or no degradation and could be considered reliable for the identification of N-terminal peptides, we next turned our attention to identifying substrates of the AtICP55 protein. It was demonstrated that Icp55 cleaves off one amino acid following MPP cleavage in yeast; and through analysis of mitochondria from *ΔScIcp55* by ChaFRADIC that substrates could be identified by looking for N-terminal peptides with an increase in abundance in the *ΔScIcp55* when compared to the wild type (Vogtle *et al.*, 2009; Venne *et al.*, 2013). Therefore, we took our list of mitochondrial N-terminal peptides and extracted peptides that had a greater than 3-fold difference when the *Δicp55* samples were compared to the wild-type samples. Both increases and decreases in ‘fold changes’ were considered. Analysing our data in this way produced a list of 106 peptides, which represent 78 different proteins (Table 1). Notably, for a number of proteins, two N-termini were identified. For example, for Oxa1 (At3g44370), the N-terminal peptide ^44^FSTPSDLDSELTR^56^ was identified, which was 60 times more abundant in the *Δicp55* mitochondria; a second N-terminal peptide ^45^STPSDLDSELTR^56^ was also identified, and was 50 times more abundant in the wild-type mitochondria. This demonstrates that AtICP55 removes the F from the N-terminus, which is why the peptide ^44^FSTPSDLDSELTR^56^ is found in far greater abundance in the *Δicp55* mitochondria compared to the wild type.

**Table 1.** Summary of the *Arabidopsis* Icp55 substrates identified

No	Accession	Gene description	Δicp55	Removed amino acid	Identified sequence
1	AT1G17290	Alanine aminotransferase	**↑↑**	L	LSSSSSSDMSASDSSSSLPVTLDTINPKVIKCEYAVR
			**↓↓**		SSSSSSDMSASDSSSSLPVTLDTINPKVIKCEYAVR
2	AT1G23800	Aldehyde dehydrogenase 2B	**↑↑**	Y	YSNLAAAVENTITPPVKVEHTQLLIGGR
			**↓↓**		SNLAAAVENTITPPVKVEHTQLLIGGR
3	AT1G32350	Alternative oxidase 1D	**↑↑**	L	LSSDTSSPVSGNNQPENPIR
			**↓↓**		SSDTSSPVSGNNQPENPIR
4	AT1G50940	Electron transfer flavoprotein alpha	**↑↑**	I	ISISITSLSR
			**↓↓**		SISITSLSR
5	AT2G14170	Aldehyde dehydrogenase 6B	**↑↑**	L	LSTSPEQSTQPQMPPR
			**↓↓**		STSPEQSTQPQMPPR
6	AT2G20360	NAD(P)-binding Rossmann-fold superfamily protein	**↑↑**	Y	YSSSLATKGVGHLAR
			**↓↓**		SSSLATKGVGHLAR
7	AT3G06050	Peroxiredoxin IIF	**↑↑**	F	FSKLAEGTDITSAAPGVSLQKAR
			**↓↓**		SKLAEGTDITSAAPGVSLQKAR
8	AT3G13860	Hsp60-3	**↑↑**	Y	YAAKDISFGIGAR
			**↓↓**		AAKDISFGIGAR
9	AT3G13930	Dihydrolipoamide acetyltransferase	**↑↑**	F	FSSSSDLPPHQEIGMPSLSPTMTEGNIAR
			**↓↓**		SSSSDLPPHQEIGMPSLSPTMTEGNIAR
10	AT3G23990	Hsp60	**↑↑**	Y	YAAKEIKFGVEAR
			**↓↓**		AAKEIKFGVEAR
11	AT3G44370	Oxa1	**↑↑**	F	FSTPSDLDSELTR
			**↓↓**		STPSDLDSELTR
12	AT3G45770	Polyketide synthase	**↑↑**	F	FSTIMSPPSKAIVYEEHGSPDSVTR
			**↓↓**		STIMSPPSKAIVYEEHGSPDSVTR
13	AT3G54980	Pentatricopeptide repeat (PPR) protein	**↑↑**	FC	FCSQSQFPKESENPSQEQR
			**↓↓**		SQSQFPKESENPSQEQR
14	AT4G08900	Arginase	**↑↑**	FT	FTSVSASSIEKGQNR
			**↓↓**		SVSASSIEKGQNR
15	AT4G35460	NADPH-dependent thioredoxin reductase B	**↑↑**	F	FSSSAVMNGLETHNTR
			**↓↓**		SSSAVMNGLETHNTR
16	AT4G36400	FAD-linked oxidases family protein	**↑↑**	F	FGSSAASLIQR
			**↓↓**		GSSAASLIQR
17	AT4G37910	Hsp70-1	**↑↑**	FC	FCSRPVGNDVIGIDLGTTNSCVSVMEGKTAR
			**↓↓**		SRPVGNDVIGIDLGTTNSCVSVMEGKTAR
18	AT5G09450	Pentatricopeptide repeat (PPR) protein	**↑↑**	YN	YNADAAIGNSLVEESEEKDDLKSR
			**↓↓**		ADAAIGNSLVEESEEKDDLKSR
19	AT5G47630	Mitochondrial acyl carrier protein 3	**↑↑**	FT	FTSEAAADGGQDQILSR
			**↓↓**		SEAAADGGQDQILSR
20	AT5G50850	Transketolase family protein	**↑↑**	Y	YAAGAKEMTVR
			**↓↓**		AAGAKEMTVR
21	AT5G55070	Dihydrolipoamide succinyltransferase	**↑↑**	F	FSSDSGDVVEAVVPHMGESITDGTLAAFLKKPGDR
			**↓↓**		SSDSGDVVEAVVPHMGESITDGTLAAFLKKPGDR
22	AT4G08390	Ascorbate peroxidase	**↑↑**	FN	FNSTTAATKSSSSDPDQLKNAR
			**↓↓**		STTAATKSSSSDPDQLKNAR
23	AT1G63940	Monodehydroascorbate reductase 6	**↑↑**	L	LVTASFANENR
			**↓↓**		VTASFANENREFVIVGGGNAAGYAAR
24	AT3G58140	Phenylalanyl-tRNA synthetase	**↑↑**	F	FSSSAAYSPPKMR
			**↓↓**		SSSAAYSPPKMR
25	AT5G04780	Pentatricopeptide repeat (PPR) protein	**↑↑**	I	ISVLASYDQEEVSPGR
			**↓↓**		SVLASYDQEEVSPGR
26	AT5G08670	ATP synthase beta	**↑↑**	Y	YATSSPASSAAPSSAPAKDEGKKTYDYGGKGAIGR
					ATSSPASSAAPSSAPAKDEGKKTYDYGGKGAIGR
27	AT4G32915	Protein coding	**↑↑**	Y	YSSDSDSSVLQPPDVAR
			**↓↓**		SSDSDSSVLQPPDVAR
28	AT1G77170	Pentatricopeptide repeat (PPR) protein	**↑↑**	F	FVTTSSSSVTPLSPQDR
			**↓↓**		VTTSSSSVTPLSPQDR
29	AT1G09410	Pentatricopeptide repeat (PPR) protein	**↓↓**	Y	STTIPPPTANVR
30	AT1G80230	Rubredoxin-like superfamily protein	**↓↓**	I	GSAAADTAVKKR
31	AT1G51965	Pentatricopeptide repeat (PPR) protein	**↓↓**	Y	ATKYVAKVTSSSPSGR
32	AT1G71210	Pentatricopeptide repeat (PPR) protein	**↓↓**	F	STFTKPSSSIAPGDFLVR
33	AT1G80550	Pentatricopeptide repeat (PPR) protein	**↓↓**	L	SVKPISNVDDAKFR
34	AT2G18520	Pentatricopeptide repeat (PPR) protein		F	FSTATGIDSQTTAYPGAITMSKAKSKLR
			**↓↓**		STATGIDSQTTAYPGAITMSKAKSKLR
35	AT2G26140	FTSH protease 4		Q	QSSYVGSFAR
			**↓↓**		SSYVGSFAR
36	AT2G31955	Cofactor of nitrate reductase	**↓↓**	F	SSSYAAHQVDQIKDNPVSDMLIDKFGR
37	AT2G38400	Alanine:glyoxylate aminotransferase 3	**↓↓**	I	SSTSQAATASVKDSDEFQAR
38	AT2G39795	Mitochondrial glycoprotein family protein	**↓↓**	Y	STAIDRISSEQTLIR
39	AT2G43360	Radical SAM superfamily protein		Y	YSSLSAASAEAER
			**↓↓**		SSLSAASAEAER
40	AT2G47510	Fumarase 1	**↓↓**	Y	STSFREERDTFGPIQVPSDKLWGAQTQR
41	AT3G02090	MPP beta	**↓↓**	Y	ASPHPILASHNHILSAPETR
42	AT3G15590	Pentatricopeptide repeat (PPR) protein		L	LSSIADAKDKGDEVVR
			**↓↓**		SSIADAKDKGDEVVR
43	AT3G22470	Pentatricopeptide repeat (PPR) protein	**↓↓**	Y	SSITEAKLSYKER
44	AT3G60510	ATP-dependent caseinolytic (Clp) protease	**↓↓**	C	SLKLTSEDLDYQVLVEGSGCSR
45	AT4G31810	ATP-dependent caseinolytic (Clp) protease	**↓↓**	F	SALPNYSASDADFEDQVLVEGKAKSR
46	AT5G08680	ATP synthase beta	**↓↓**	Y	STSSPANSAAPSSAPAKDEGKKTYDYGGKGAIGR
47	AT5G15280	Pentatricopeptide repeat (PPR) protein	**↓↓**	F	STSSPASSSSSSLGNDSAIPR
48	AT5G23140	Nuclear-encoded CLP protease P7	**↓↓**	Y	SLIPMVIEHSSR
49	AT5G60960	Pentatricopeptide repeat (PPR) protein	**↓↓**	F	SSETNAESESLDSNEIALSFSKELTGNPDAESQTISQR
50	AT3G02780	Isopentenyl pyrophosphate isomerase 2		F	FSGTAMTDTKDAGMDAVQR
			**↓↓**		SGTAMTDTKDAGMDAVQR
51	AT3G59760	O-acetylserine (thiol) lyase isoform C	**↓↓**	F	ADGSERDPSVVCEAVKR
52	AT3G49240	Pentatricopeptide repeat (PPR) protein	**↓↓**	M	SFATQEEAAAERR
53	AT1G24880	UDP-3-O-acyl N-acetylglycosamine deacetylase	**↑↑**	Y	YSSAASSPTVSLNPSGR
54	AT1G48030	Mitochondrial lipoamide dehydrogenase 1	**↑↑**	F	FASSGSDENDVVIIGGGPGGYVAAIKASQLGLKTTCIEKR
					ASSGSDENDVVIIGGGPGGYVAAIKASQLGLKTTCIEKR
55	AT1G49650	Alpha/beta-Hydrolases superfamily protein	**↑↑**	I	ICSHSSSEIISEHPPFVR
56	AT1G54220	Dihydrolipoamide acetyltransferase	**↑↑**	F	FSSGSDLPPHQEIGMPSLSPTMTEGNIAR
57	AT1G65290	Mitochondrial acyl carrier protein 2	**↑↑**	F	FSEEVRGSFLDKSEVTDR
					SEEVRGSFLDKSEVTDR
58	AT2G27730	Copper ion binding	**↑↑**	F	FSSGKVLSEEER
					SSGKVLSEEER
59	AT2G44620	Mitochondrial acyl carrier protein 1	**↑↑**	F	FSSHDDHLSR
60	AT3G03070	NADH-ubiquinone oxidoreductase-related;	**↑↑**	F	FSVATTQLGIPTDDLVGNHTAKWMQDR
					SVATTQLGIPTDDLVGNHTAKWMQDR
61	AT3G15020	Lactate/malate dehydrogenase family protein	**↑↑**	F	FASESVPDR
62	AT3G15640	Rubredoxin-like superfamily protein	**↑↑**	F	FSSDSVETPATKKVEDVMPIATGHEKEELEAELEGR
					SSDSVETPATKKVEDVMPIATGHEKEELEAELEGR
63	AT3G17240	Lipoamide dehydrogenase 2	**↑↑**	F	FASSGSDDNDVVIIGGGPGGYVAAIKAAQLGLKTTCIEKR
					ASSGSDDNDVVIIGGGPGGYVAAIKAAQLGLKTTCIEKR
64	AT3G30775	Methylenetetrahydrofolate reductase family protein	**↑↑**	F	FSSIPTSDLLR
65	AT3G48000	Aldehyde dehydrogenase 2B	**↑↑**	F	FGTSSAAAEEIINPSVQVSHTQLLINGNFVDSASGKTFPTLD
					GTSSAAAEEIINPSVQVSHTQLLINGNFVDSASGKTFPTLD
66	AT4G26910	Dihydrolipoamide succinyltransferase	**↑↑**	F	FSAETGDTVEAVVPHMGESITDGTLATFLKKPGER
					SAETGDTVEAVVPHMGESITDGTLATFLKKPGER
67	AT4G35850	Pentatricopeptide repeat (PPR) protein	**↑↑**	F	FASSPEEIAKR
					ASSPEEIAKR
68	AT5G08300	Succinyl-CoA ligase, alpha subunit;	**↑↑**	F	FASDPHPPAAVFVDKNTR
					ASDPHPPAAVFVDKNTR
69	AT5G09590	Hsp70-2	**↑↑**	F	FSSKPAGNDVIGIDLGTTNSCVAVMEGKNPKVIENAEGAR
					SSKPAGNDVIGIDLGTTNSCVAVMEGKNPKVIENAEGAR
70	AT5G23250	Succinyl-CoA ligase	**↑↑**	F	FGTTPPPPAAVFVDKNTR
					GTTPPPPAAVFVDKNTR
71	AT5G67590	NADH-ubiquinone oxidoreductase-related	**↑↑**	F	FATDAVVESDYKR
					ATDAVVESDYKRGEIGKVSGIPEEHLSR
72	AT5G64050	Glutamate tRNA synthetase	**↑↑**	F	FAVVACSTPVNNGGSVR
					AVVACSTPVNNGGSVR
73	AT1G53240	Lactate/malate dehydrogenase family protein	**↑↑**	F	FSSGSVPER
74	AT1G47720	Primosome PriB/single-strand DNA-binding	**↑↑**	F	FSDGESAVYHHAR

Up (↑↑) and down (↓↓) regulation of peptides in the Δicp55 mitochondria (only peptides with at least a 3-fold change were considered). The identified peptide sequence is shown, as well as the amino acid (or amino acids) removed by AtICP55.

While over half of the proteins with two identified N-termini showed differential abundances between wild type and *Δicp55* mitochondria, we also included proteins when only one of the two identified N-termini displayed a greater than 3-fold difference between *Δicp55* and the wild type (Table 1). For example, Biotin synthase (At2g43360) had two different N-terminal peptides identified ^26^YSSLSAASAEAER^38^ and ^27^SSLSAASAEAER^38^, with only the second peptide downregulated 100 times in the *Δicp55* mitochondria. In other cases only one N-terminal peptide was identified and was included based on two different criteria: first, if the identified N-terminal peptide was upregulated in the *Δicp55* mitochondria and the very N-terminal amino acid was considered unstable according to the N-end rule (Bachmair *et al.*, 1986; Varshavsky, 2011); second, if the identified N-terminal peptide was downregulated in the *Δicp55* mitochondria and the last amino acid cleaved off was unstable (Bachmair *et al.*, 1986; Varshavsky, 2011). These proteins were also included as ICP55 substrates in *Arabidopsis* mitochondria. Together this produced a total of 74 proteins, which are putative substrates for the AtICP55 (Table 1).

In a second round of analyses we decreased our criteria and included peptides that only showed a difference between wild-type and *Δicp55* samples of between 1.5- and 3-fold. This identified a further 14 potential substrates for the *Arabidopsis* ICP55 protein (Table 2). Putting these results together, we have identified 88 potential substrates for the *Arabidopsis* ICP55 protein.

**Table 2.** Further Possible *Arabidopsis* Icp55 substrates

No	Accession	Gene description	Δicp55	Removed amino acid	Identified sequence
75	AT1G22800	*S*-adenosyl-L-methionine-dependent methyltransferases	**↓↓**	F	STEGAYGGDGEFQQNSSKVKIFDRDLKR
76	AT1G56690	Pentatricopeptide repeat (PPR) superfamily protein	**↑↑**	Y	YLTSTGVNCSFEISR
77	AT1G72330	Alanine aminotransferase 2	**↓↓**	F	SSTSEMSASDSTSSLPVTLDSINPKVLKCEYAVR
78	AT2G30920	Coenzyme Q 3	**↓↓**	F	STSDTDASAASFSSSHPKIQTLEGKASNKSR
79	AT3G17465	Ribosomal protein L3	**↓↓**	F	SSDTGLMDGGGSDIIGAQTR
80	AT3G56030	Pentatricopeptide repeat (PPR) superfamily protein	**↓↓**	F	STVNPNPTASPGR
81	AT4G11120	Translation elongation factor Ts	**↓↓**	F	SSEAPPAVSDQMSLIKQLR
82	AT4G28630	ABC transporter of the mitochondrion 1	**↓↓**	F	STSTSTPNQDQTKTASSKKILR
83	AT5G03905	Iron-sulphur cluster biosynthesis family protein	**↓↓**	F	SSASAIKEASSSSSSQPESSSNDVVHLSDNCIR
84	AT5G08530	51kDa subunit of complex I	**↓↓**	F	STQAASTSTTPQPPPPPPPPEKTHFGGLKDEDR
85	AT5G15010	Pentatricopeptide repeat (PPR) superfamily protein	**↓↓**	F	STSIADSEQVGFTR
86	AT5G66760	Succinate dehydrogenase 1-1	**↓↓**	F	STGSTDTRSSYTIVDHTYDAVVVGAGGAGLR
87	AT2G15690	Pentatricopeptide repeat (PPR) superfamily protein	**↓↓**	L	STSAAANDYHQNPQSGSPSQHQRPYPPQSFDSQNQTNTNQR
88	AT3G54660	Glutathione reductase	**↓↓**	F	SVCASTDNGAESDRHYDFDLFTIGAGSGGVR

Up (↑↑) and down (↓↓) regulation of peptides in the Δicp55 mitochondria (peptides with between a 1.5- and 3-fold change). The identified peptide sequence is shown, as well as the amino acid (or amino acids) removed by AtICP55.

**Fig. 2.**
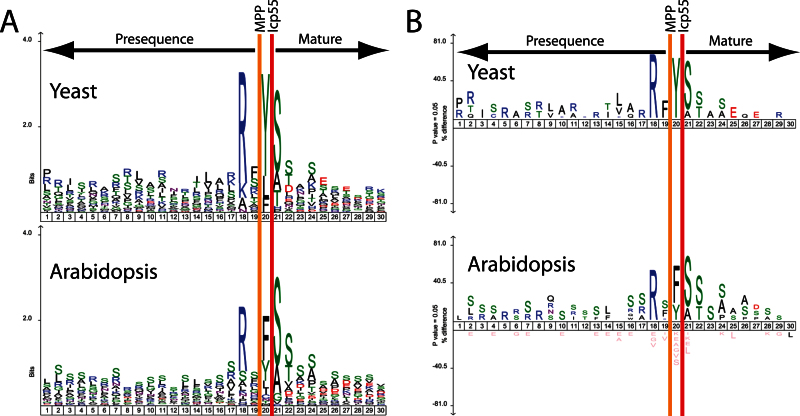
SequenceLogo and Icelogo analysis of the cleavage sites of both yeast and *Arabidopsis* ICP55 proteins. (A) Relative frequency of amino acids in presequences (up to 20 amino acids of the C-terminal segment) and the first 10 amino acids of the mature proteins identified in ICP55 substrates from both yeast and *Arabidopsis*. (B) The same sequences as in (A), but instead of relative frequency it displays an Icelogo. Icelogo displays the over- and under-represented amino acids compared to each organism’s reference set. The display is in terms of a percentage difference.

To analyse the substrate specificity of the AtICP55 protein, sequenceLogo and iceLogo plots were generated for the 20 amino acids upstream of the wild-type determined N-terminus and 10 amino acids downstream (Fig. 2). What can be clearly seen is that AtICP55 has a consensus cleavage motif of RX(F/Y/I/L)↓(S/A)(S/T). Also, the mainly unstable amino acids F, Y,and L are the most abundant amino acids removed by AtICP55 in a –3R environment. Interestingly, the AtICP55 can also remove other amino acids, including I, Q, and M. I and Q are understandable as they are potentially unstable as N-termini in plants (Graciet and Wellmer, 2010). In comparison to the known substrate specificity of the yeast Icp55, the AtICP55 displays a remarkably high similarity (Fig. 2). This is in contrast to the fact that yeast and *Arabidopsis* have evolved separately for over a billion years since they last shared a common ancestor.

AtICP55 also appears to be able to cleave some proteins twice. For example, the mitochondrial acyl carrier protein 3 (At5g47630) had three different N-termini identified: ^38^FTSEAAADGGQDQILSR^54^, upregulated 100-fold in *Δicp55* mitochondria; ^39^TSEAAADGGQDQILSR^54^, downregulated 10-fold in *Δicp55* mitochondria; and ^40^SEAAADGGQDQILSR^54^, downregulated 100-fold in *Δicp55* mitochondria. This means that the mitochondrial acyl carrier protein is first cleaved by MPP and then subsequently cleaved by AtICP55 either twice or by some unknown protease, to finally leave the stable amino acid S at its N-terminus. Taking all this together, AtICP55 protein removes unstable amino acids from the N-termini of mitochondrial proteins after MPP cleavage.

### Identification of AtOCT1 substrates

**Fig. 3.**
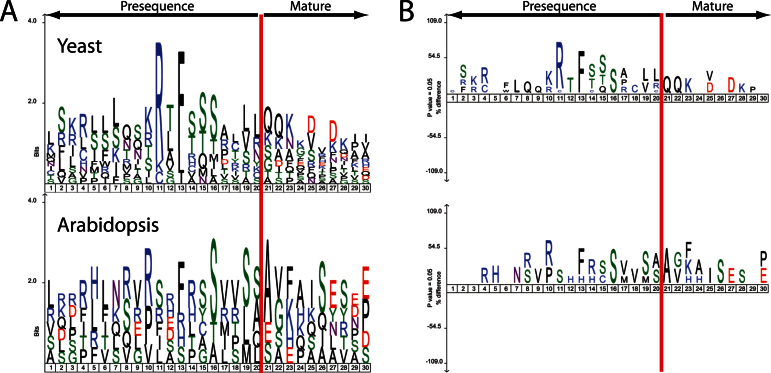
Sequencelogo and Icelogo analysis of the cleavage sites of both yeast and *Arabidopsis* OCT1 proteins. (A) Relative frequency of amino acids in presequences (up to 20 amino acids of the C-terminal segment) and the first 10 amino acids of the mature proteins identified in OCT1 substrates from both yeast and *Arabidopsis*. (B) The same sequences as in (A) but instead of relative frequency it displays an Icelogo.

After determining the AtICP55 cleavage sites and substrate specificity we next turned to AtOCT1, which should remove eight amino acids following MPP cleavage (Vogtle *et al.*, 2011; Teixeira and Glaser, 2013). Studying the *Arabidopsis* Oct1 is interesting because in plant mitochondrial presequences, no –10R motif has been identified to date, though the *Arabidopsis* and yeast Oct1 proteins are clearly related (Huang *et al.*, 2009; Teixeira and Glaser, 2013). *Δoct1-1* and *Δoct1-2* mitochondria were analysed by ChaFRADIC in the same way as for the *Δicp55* mitochondria. However, in contrast to *Δicp55*, only 12 peptides were identified in the *Δoct1* mitochondrial samples, covering seven proteins that have a greater than 3-fold difference compared to the wild type (Table 3). To see if the proteins identified contained the characteristic –10R motif we compared sequenceLogo and iceLogo plots of 20 amino acids upstream and 10 amino acids downstream of the known yeast Oct1 substrates and the potential substrates identified from the ChaFRADIC *Arabidopsis* data (Fig. 3). What is evident is that the yeast substrates clearly show the –10R motif, whereas the *Arabidopsis* samples show no real sequence motif. To obtain independent evidence that these are clear OCT1 substrates we used *in vitro* imports into isolated mitochondria and looked for size shifts in the imported mature bands.

**Table 3.** Summary of the *Arabidopsis* Oct1 substrates identified

No.	Accession	Gene description	Δoct1	Identified sequence
1	AT1G14610	Valyl-tRNA synthetase	1↓↓, 2↓↓	ESEKKILTEEELER
2	AT1G80550	Pentatricopeptide repeat (PPR) protein	1↓↓, 2↓↓	SVKPISNVDDAKFR
3	AT2G32230	Proteinaceous RNase P1 (PRORP1)	1↓↓, 2↓↓	AAKQSAASPSENLSR
4	AT3G22310	Mitochondrial RNA helicase 1 (PMH1)	2↑↑	FHVKSVPSEFR
5	AT3G22330	Mitochondrial RNA helicase 2 (PMH2)	2↑↑	IHFQSGPLDFR
			1↑↑, 2↑↑	MVSQAGFAISESSER
			1↑↑, 2↑↑	VSQAGFAISESSERR
			1↑↑, 2↑↑	SQAGFAISESSERR
			1↓↓, 2↓↓	AGFAISESSERR
			1↓↓, 2↓↓	GFAISESSER
6	AT5G52840	B13 NADH complex	1↓↓, 2↓↓	AKVKQTTGIVGLDVVPNAR
7	AT3G48110	glycine-tRNA synthetase	2↓↓	AVHHQSYRNPDDDVTR

Up (↑↑) and down (↓↓) regulation of peptides in the Δoct1 mitochondria (only peptides with at least a 3-fold change were considered). The number 1 or 2 indicates which of the two Δoct1 mutants the different regulation comes from.

**Fig. 4.**
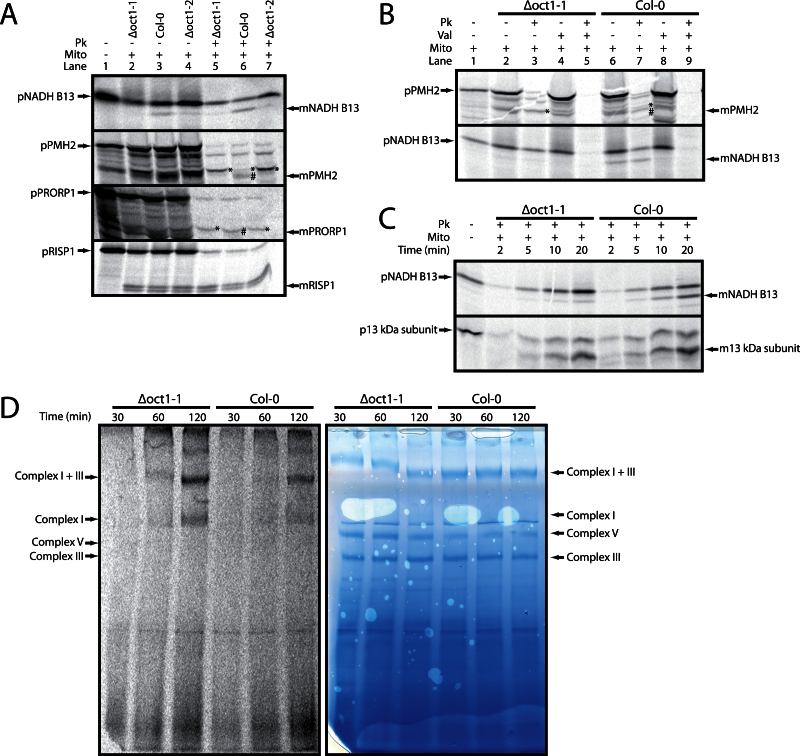
Confirmation of AtOCT1 substrate cleavage and consequences of the lack of processing of NADH B13. (A) *In vitro* import analysis of potential AtOCT1 substrates into isolated mitochondria from the wild type (Col-0) and the two Δoct1 lines (designated 1 and 2). Lanes: 1, precursor protein only; 2, precursor protein incubated with Δoct1-1 mitochondria under conditions that support import into mitochondria; 3, as lane 2 but wild-type mitochondria; 4, as lane 2 but Δoct1-2 mitochondria; 5–7, as lanes 2–4 but with proteinase K added after incubation of the precursor with mitochondria. (B) *In vitro* imports of PMH2 (At3g22330) and NADH B13 (At5g52840) into wild type and Δoct1-1 demonstrating that the import bands are dependent on the membrane potential. Lanes 1–5 are the same as from Fig. 1B but with Δoct1-1 mitochondria. Lanes 6–9 are the same as lanes 2–5 but with wild-type mitochondria. (C) *In vitro* imports into isolated Δoct1-1 and wild-type mitochondria for NADH B13 (At5g52840) and 13 kDa subunit (At3g03070) analysed over time (2, 5, 10, and 20min). (D) Assembly of NADH B13 (At5g5280) into Complex I and super complex I + III analysed by *in vitro* imports and BN-PAGE over time (30, 60, and 120min) for Δoct1-1 and wild-type mitochondria. Mito, mitochondria; Pk, proteinase K; Val, valinomycin; min, minutes; p, precursor protein; m, mature protein; *, the Oct1 intermediate product (MPP-only cleavage); #, fully processed MPP and Oct1 cleaved product.

The proteins chosen for analysis were the valyl-tRNA synthetase (At1g14610), proteinaceous RNase P1 (PRORP1, At2g32230), mitochondrial RNA helicase 2 (PMH2, At3g22330), B13 NADH complex protein (At5g52840), and the Rieske iron sulfur protein from complex III (Rieske FeS, At5g13430), as it is a model substrate for yeast Oct1. *In vitro* import assays were carried out using radiolabelled precursors; these were incubated with mitochondria isolated from the wild type, *Δoct1-1*, and *Δoct1-2*. Following import experiments, half of the reactions were treated with proteinase K to remove non-imported or bound precursor proteins. For the protein valyl-tRNA synthetase, no import into isolated mitochondria was observed (data not shown). However, for PMH2, B13, PRORP1, and Rieske FeS mitochondrial import was observed, indicated by the presence of protease-protected bands following the addition of proteinase K (Fig. 4A). Interestingly, the Rieske FeS protein did show two processed bands after import, but both were still evident in the *Δoct1* mutants, indicating that it is processed twice, but not by OCT1, in *Arabidopsis* (Fig. 4A). Both PMH2 and PRORP1 demonstrated good import into both wild type and *Δoct1* mutant mitochondria, but in both *Δoct1* mutant samples the imported band ran at a higher apparent molecular weight, indicating a lack of processing by OCT1 (Fig. 4A). This supports the ChaFRADIC data showing that the wild type N-termini of both of these proteins are less abundant in the mutant mitochondria. For B13 an imported, protease-resistant band of a lower molecular weight was only observed in mitochondria from wild type (Fig. 4A). In contrast, in the *Δoct1* mitochondria, only an unprocessed band was detected. However, this unprocessed band was also protease resistant, while the precursor itself was susceptible to protease treatment under non-permissive import conditions (see below). This is interesting because it appears as though the B13 presequence is not cleaved by MPP, but exclusively by OCT1. Again this would support the ChaFRADIC data, with the processed N-termini being reduced in abundance in the *Δoct1* mitochondrial samples.

To follow up the initial import experiments it was decided to import B13 and PMH2 into isolated mitochondria, but also in the presence of valinomycin. Valinomycin is an ionophore that dissipates the membrane potential of mitochondria, which is required when importing proteins across the inner membrane. No protease-protected proteins were detected in the reactions containing valinomycin, while in its absence, a processed mature form was detectable (Fig. 4B). Again, the size shift between wild-type and *Δoct1-1* mitochondria was evident. For B13, both the precursor protein and the processed protein are protease resistant in the absence of valinomycin, but susceptible to protease treatment in its presence (Fig. 4B). Therefore, both forms are imported into mitochondria. Again, the processed band was only present in the wild type.

B13 is a subunit of complex I, and one of the roles of Oct1 cleavage in yeast is thought to be control of complex assembly. The assembly of B13 into complex I was monitored using *in vitro* imports. It can clearly be seen that the processed mature band accumulated in the wild type, but only the precursor accumulated in the *Δoct1-1* mitochondria over time (Fig. 4C). We used an additional complex I subunit, the so-called 13kDa subunit (At3g03070) as a control. It shows very similar import behaviour in both wild-type and *Δoct1-1* mitochondria (Fig. 4C). When the assembly of B13 into complex I and the super complex I + III was analysed using BN-PAGE, the assembly rate was higher in the mutant than in the wild type, evidenced by the stronger labelling of the complexes in the mutant sample (Fig. 4D). This could not be due to unequal loading as the Coomassie stain of the same gel shows that the protein bands have equal intensity. This means that B13 can be imported and assembled into mitochondria and complex I in the absence of processing. It is tempting to speculate that the OCT1 cleavage of B13 occurs following assembly. A similar observation was also made with Rieske FeS from yeast, which was shown to be imported and assembled without Oct1 cleavage (Nett and Trumpower, 1999).

### Properties of *Arabidopsis* mitochondrial presequences

After identifying substrates for AtICP55 and AtOCT1 using ChaFRADIC, we re-analysed our data to identify the N-terminal sequences for as many mitochondrial proteins as possible (Supplementary Table S2). By combining all samples and extracting only mitochondrial proteins and their most N-terminal peptide (not including peptides upregulated in the *Δoct1* and *Δicp55* samples) we could identify the N-terminal sequences for 179 proteins (Supplementary Tables S3 and S4). This includes both processed (144) and unprocessed (35) mitochondrial proteins. In order to confirm that the sequences obtained were accurate, we compared them to previously obtained N-terminal sequences which were obtained by semi-trypic peptides (Huang *et al.*, 2009) and Edmann sequencing (Kruft *et al.*, 2001). In total, 52 of the proteins identified in this study had been previously identified (Supplementary Table S5). Out of these 52 proteins, 40 had identical N-termini in this study and the previous studies. For the 12 N-termini that differed between this study and the previous studies, the majority are only differentiated by a single amino acid, and in some of these cases it would appear that the difference is the un-cleaved Icp55 N-terminal peptide. For example, the Clp protease (At5g23140) had the previously identified N-terminal peptide ^30^YSLIPMVIEHSSR^42^ (Huang *et al.*, 2009); however, in this study, the N-terminal peptide ^31^SLIPMVIEHSSR^42^ was identified. This was also downregulated in the *Δicp55* mitochondria, indicating that it was a substrate of AtICP55, most likely cleaving the Y after MPP cleavage (Table 1). Another difference was for Aconitase 3, which had previously been identified as having a cleaved presequence (Huang *et al.*, 2009), and was identified in this study as having no presequence. These small differences may reflect the advantages or disadvantages of using different methods of N-terminal identification.

By combining the N-termini identified in this study (144) with other identified N-terminal sequences (32) (Huang *et al.*, 2009), a list of 176 N-terminal peptides can be assembled. These can then be used to determine the mitochondrial presequence for each protein, creating the largest data set of plant mitochondrial presequences from one species known to date. To analyse the properties of this data set, data sets comprising the identified yeast mitochondrial presequences (a total of 416 presequences; Vogtle *et al.*, 2009), chloroplast targeting signals (154 targeting sequences; Zybailov *et al.*, 2008; Bienvenut *et al.*, 2012), and dual-targeted proteins that are targeted to mitochondria and chloroplasts with identified N-termini (a total of 26 proteins; Carrie and Whelan, 2013) were compiled (Supplementary Table S4). These four data sets were then compared using four different properties: presequence length, net charge, amino acid content, and cleavage site motifs.

**Fig. 5.**
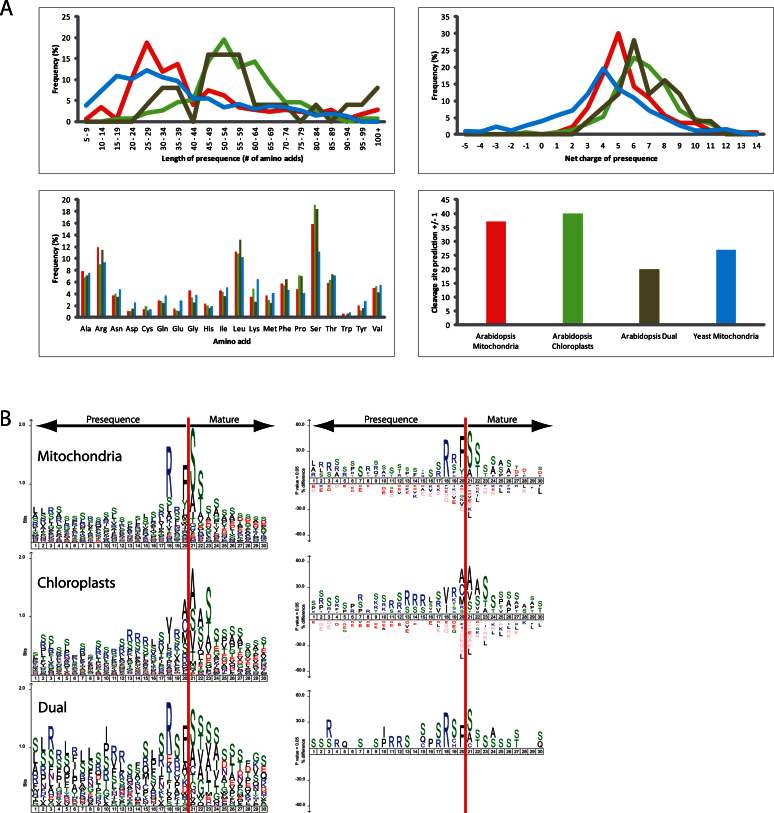
Analysis of mitochondrial presequences from yeast and *Arabidopsis*, transit peptides from *Arabidopsis* chloroplasts, and dual-targeted proteins from *Arabidopsis*. (A) The mitochondrial presequences from *Arabidopsis* and yeast, transit peptides from *Arabidopsis* chloroplast proteins, and targeting signals from *Arabidopsis* dual-targeted proteins were analysed for length in amino acids, overall net charge, frequency percentage of individual amino acids, and percentage of correct prediction of the cleavage site (+/– 1 amino acid) by the program TargetP. Blue, yeast mitochondria; red, *Arabidopsis* mitochondria; green, *Arabidopsis* chloroplasts; brown, *Arabidopsis* dual-targeted proteins. (B) SequenceLogo and Icelogo analysis comparing the cleavage sites of *Arabidopsis* mitochondrial presequences, *Arabidopsis* chloroplastic transit peptides, and the ambiguous targeting signals of dual-targeted proteins from *Arabidopsis*. Dual-targeted proteins are targeted to both mitochondria and chloroplasts.

Firstly, it was found that the lengths of *Arabidopsis* mitochondrial presequences ranged from 7 to 117 amino acids, with an average of 43.3 amino acids (Fig. 5). This is significantly longer than the yeast mitochondrial presequences (*P* = 0.00127), which have a similar range from 6 to 94 amino acids, with an average of 37.1 amino acids (Fig. 5). As has been demonstrated previously, chloroplast transit peptides are significantly longer than mitochondrial presequences (*P* = 1.5E-9), with a range from 15 to 152 amino acids and an average length of 56 amino acids (Fig. 5). While the number may be small (25), it was thought that including dual-targeted proteins in these analyses might prove interesting. Dual-targeted presequences were determined to be on average 58.8 amino acids long, with a range of 27 to 101 amino acids. Therefore, dual-targeted presequences are not significantly different in length to chloroplast transit peptides (*P* = 0.3), but are significantly longer than mitochondrial presequences (*P* = 0.0016) (Fig. 5).

Secondly, the presequences were analysed for their net charge. *Arabidopsis* mitochondrial presequences were determined to have an average positive charge of 5.6, which is significantly more positive (*P* = 1.2E-7) than yeast mitochondrial presequences, which displayed an average of 3.8 (Fig. 5). Overall, *Arabidopsis* chloroplast transit peptides are more positive with an average charge of 6.6, which is significantly more than for mitochondrial presequences (*P* = 0.00461)(Fig. 5). Interestingly, dual-targeted proteins appear to have a higher average charge of 6.9 when compared to chloroplast transit peptides and mitochondrial presequences. However, the majority appear to fall somewhere in between mitochondrial presequences and chloroplast transit peptides(Fig. 5).

Thirdly, the amino acid frequencies in all of the samples were analysed. Surprisingly, no widespread differences were observed, with most amino acids having similar frequencies in all samples. Yeast mitochondrial presequences appear to be slightly more enriched for Asp, Gln, Glu, and Lys compared to *Arabidopsis* mitochondrial presequences, chloroplast transit peptides, and dual-targeted proteins. *Arabidopsis* mitochondrial presequences and dual-targeting signals have slightly more Arg residues. Chloroplast transit peptides and dual-targeting signals have more Ser residues, and *Arabidopsis* has more Ser residues in mitochondrial presequences when compared to yeast.

Finally, the subcellular targeting prediction program TargetP was tested for its ability to predict the correct cleavage site in the four data sets. TargetP predicts both MPP and SPP cleavage sites (Emanuelsson *et al.*, 2007). We allowed the program a plus or minus one accuracy. Interestingly, TargetP proved better at correctly predicting *Arabidopsis* mitochondrial presequences and chloroplast transit peptides with 37 and 40% accuracy for both data sets, respectively. TargetP performed worst at predicting the cleavage sites of dual-targeted proteins, with only 20% accuracy. However, it performed slightly better with yeast mitochondrial presequences, with a 27% accuracy. Overall this low accuracy demonstrates that more work is required to identify the correct cleavage sites experimentally, so that programs like TargetP can be improved.

### Cleavage sites of dual-targeted proteins

The data set of 26 dual-targeted presequences in this study comprises almost 25% of the known dual-targeted proteins in *Arabidopsis* (currently 112 proteins: Carrie and Small, 2013; Carrie and Whelan, 2013), thus representing a reliable source for this analysis. Previously, most data sets had used arbitrary numbers, such as the first 70 amino acids of dual-targeted proteins, to analyse the presequences. However, here not only are the presequences available, but the N-terminal peptide sequences can be used to identify the cleavage sites for dual-targeted proteins. Using the same four data sets as above, sequenceLogo and iceLogo plots of the 20 upstream amino acids and 10 downstream amino acids of the final cleavage sites were generated (Fig. 5). We observed that *Arabidopsis* mitochondrial presequences contain the two classic –3 and –2R cleavage motifs, with –3R being more prevalent, giving an overall cleavage site motif of RX(F/Y/)↓(S/A)(S/T). For the *Arabidopsis* chloroplast transit peptides, the cleavage site motif is not as conserved as for mitochondrial presequences; nevertheless, a consensus motif of (V/I)X(A/C/M/S)↓(A/S/V)(S/A/V/)(S/T/A/V) can be deduced. Surprisingly, the cleavage analysis of *Arabidopsis* dual-targeted proteins displayed a sequence motif of RX(F/L)↓(S/A)(S/T/V/A), which is remarkably similar to the mitochondrial cleavage motif. It is also similar to the cleavage motif of AtICP55; in fact, seven of the dual-targeted proteins were identified as AtICP55 substrates (Tables 1 and 2). While the cleavage motif for dual-targeted proteins may resemble the mitochondrial cleavage motif, this may just reflect where the N-terminal sequences were obtained from. The majority were either from this study (isolated mitochondria) or from a previous mitochondrial publication (20 proteins in total; Huang *et al.*, 2009); only a small number came from chloroplast samples (5 proteins; Zybailov *et al.*, 2008). This is especially evident when considering the examples of peptide deformylase (At1g15390) and the Asp-tRNA synthetase (At4g17300), which have had different N-termini identified depending on the sample analysed. Therefore, it is possible that more dual-targeted proteins have slightly different cleavage sites within the two organelles. However, *in vitro* import experiments suggest that most dual-targeted proteins are cleaved to the same size, indicating either identical cleavage sites or cleavage sites that are very close together (Chew *et al.*, 2003; Berglund *et al.*, 2009; Ge *et al.*, 2014).

## Discussion

We have shown that *Arabidopsis* mitochondria contain a homologue of the yeast intermediate peptidase Icp55. Not only are the *Arabidopsis* and yeast Icp55 proteins similar at a sequence level, they also display remarkably similar processing sites. SequenceLogo plots of the yeast Icp55 processing sites display a consensus processing motif of RX(Y/L/F)↓(S/A)(S/T), with the *Arabidopsis* ICP55 displaying an almost identical consensus motif of RX(F/Y/I/L)↓(S/A)(S/T). The only major differences between the yeast and *Arabidopsis* homologues is that the AtICP55 is responsible for processing many more mitochondrial proteins and can cleave off more amino acids than just Y, L, and F. We have also identified 88 potential substrates for AtICP55, compared to the 52 substrates identified in yeast (Vogtle *et al.*, 2009; Venne *et al.*, 2013). The difference is even more pronounced when considering that in yeast mitochondria 416 proteins have had their processing sites determined, while in *Arabidopsis* mitochondria only 176 processing sites have been determined. This means that 52% of *Arabidopsis* mitochondrial proteins with cleavable presequences are further processed by ICP55, compared with only 12% in yeast. This greater number of ICP55 substrates in *Arabidopsis* is also probably the reason why, when plant mitochondrial processing sites have been analysed in the past, the –3R motif has been by far the most abundant (Glaser *et al.*, 1998; Huang *et al.*, 2009; Teixeira and Glaser, 2013).

The fact that yeast and *Arabidopsis* ICP55 proteins have such similar processing sites raises interesting questions about the evolution of ICP55, since yeast and *Arabidopsis* have been evolving separately for ~1 billion years (Yoon *et al.*, 2004). It has been speculated that Icp55 probably evolved from a bacterial amino peptidase based on its similarity to the bacterial APP family (aminopeptidase P family) (Vogtle *et al.*, 2009; Teixeira and Glaser, 2013). However, the APP cleavage motif is reported as being X↓P, with X being the N-terminal residue cleaved off (Wilce *et al.*, 1998). Obviously ICP55 has developed a far broader substrate specificity, but as demonstrated here, it is almost identical between *Arabidopsis* and yeast. This is most likely because ICP55 evolved early on in the evolution of mitochondria. It can easily be envisaged that as more and more proteins have evolved to contain mitochondrial presequences, not all will have stable N-termini after MPP cleavage. In fact, evidence for an ICP55-like protein in even further distantly related organisms can be found. In a recent study of the mitochondrial proteome of the amoeboid protist *Acanthamoeba castellanii*, RX(Y/L/F)↓(A/S/T)(T/S) was also identified for mitochondrial processing sites, which is again almost identical to the yeast and *Arabidopsis* ICP55 cleavage motif (Gawryluk *et al.*, 2014). Blast searches of *A. castellanii* also revealed the presence of a protein with high similarity to ScIcp55 and AtICP55 (data not shown). Therefore, it can be concluded that ICP55 probably evolved early on in mitochondrial evolution, but in some cases, such as in *Arabidopsis*, it has also developed a slightly broader substrate specificity.

Since ICP55 probably arose early in mitochondrial evolution, and the cleavage sites between distantly related organisms are almost identical, it can be concluded that ICP55 has the same function in all organisms. That is to covert unstable N-termini produced by MPP cleavage into stable N-termini by the removal of the unstable amino acid in accordance to the N-end rule (Vogtle *et al.*, 2009). The N-end rule was proposed almost 30 years ago by Varshavsky and colleagues, who demonstrated that the stability of a protein is dependent upon the identity of the N-terminal amino acid (Bachmair *et al.*, 1986). Since these initial experiments it was further discovered that the N-end rule is found in both prokaryotes and eukaryotes, including plants (Graciet and Wellmer, 2010; Varshavsky, 2011). Therefore, since both ScIcp55 and AtICP55 remove similar amino acids from the N-terminus, it can be inferred that the N-end rule is also utilized within plant mitochondria.

This study also demonstrated that the *Arabidopsis* ICP55 protein is targeted to both the nucleus and mitochondria through alternative splicing. This is interesting because the yeast Icp55 protein has also been shown to target both the nucleus and mitochondria through an as yet unknown mechanism (Naamati *et al.*, 2009). It is also unclear exactly what the role of ScIcp55 is in the nucleus, although one suggestion is for the processing of Nfs1, which is a cysteine desulfurase involved in iron sulfur cluster assembly and shown to be located in both mitochondria and the nucleus (Naamati *et al.*, 2009). It is tempting to speculate that a similar mechanism may also be carried out in *Arabidopsis*. While the *Arabidopsis* Nfs1 (At5g65720) was identified in this study, it was not identified as an AtICP55 substrate by ChaFRADIC; closer analysis of the identified N-terminus showed that the processing site for AtNfs1 is a –3R with the last L amino acid before the N-terminus. This means that AtNfs1 is also possibly processed by ICP55. As yet, there is no experimental evidence to suggest that AtNfs1 is located in the nucleus, even though it contains the same nuclear targeting as yeast Nsf1, suggesting that it is located in the nucleus as well as mitochondria. Therefore, more work on *Arabidopsis* nuclear samples is required to determine the exact role of AtICP55 in the nucleus.

While AtICP55 was shown to have an almost identical processing-site motif compared to yeast, the same cannot be said of AtOCT1. In fact, the small number of AtOCT1 substrates identified does not allow us to deduce a clear consensus cleavage motif. What was observed was that AtOCT1 substrates do not contain the –10R motif found in yeast Oct1 substrates. This is not altogether surprising considering the ScOct1 –10R motif has never been identified in *Arabidopsis* or other plant mitochondrial proteins (Huang *et al.*, 2009; Teixeira and Glaser, 2013). Interestingly, not only does AtOCT1 process presequences after MPP cleavage, but it also appears that it can process presequences without MPP. Such is the case of the B13 subunit of complex I; in the *Δoct1* mutants it was not processed at all. The B13 subunit of complex I is a conserved subunit found in the peripheral arm of complex I from plants, humans, bovine spp., and *Yarrowia lipolytica* (Braun *et al.*, 2014). Interestingly, in bovine spp. the B13 subunit does not contain a cleavable presequence, and in alignment with plant proteins, all plant B13 proteins contain a short presequence as in *Arabidopsis* (Hirst *et al.*, 2003). The exact reason for this short presequence is unclear; however, when looking at the sequences of plant B13 proteins, almost all have an F as their second amino acid, suggesting that if only the first M was cleaved then this would expose an F as the N-terminal amino acid, which would be unstable (Bachmair *et al.*, 1986; Graciet and Wellmer, 2010; Varshavsky, 2011). It is also possible that plant B13 proteins require a short targeting signal to avoid targeting to the chloroplasts. Interestingly, as shown by the *in vitro* imports, cleavage by OCT1 is not a requirement for assembly of B13. This leads to the possibility that AtOCT1 cleaves B13 after its insertion into complex I. Something similar has been proposed for the Oct1 processing of the Rieske FeS protein from yeast complex III (Nett and Trumpower, 1999). In yeast it has been proposed (Nett and Trumpower, 1999) that the Rieske FeS protein is first processed by MPP, subsequently inserted into complex III, and only then finally processed into its mature form by Oct1, which is similar to what we propose for the plant B13 protein.

Looking at the other substrates for OCT1, no clear picture emerges as to why they are cleaved. In yeast, it was demonstrated that like Icp55, Oct1 removes unstable amino acids from the N-terminus. By analysing the N-terminal peptides obtained for the two PMH proteins, which are almost identical to each other, it can be speculated that MPP first cleaves the presequence and then OCT1 cleaves another 17 amino acids from the N-terminus. Since peptides were also identified from sequences between these two locations, it is conceivable that this processing is not performed in one step, but that OCT1 cleaves sequentially, and hence the different N-terminal peptides found. The reason for OCT1 processing in this case is to remove either an F or an I from the MPP-generated N-termini. So, B13 could also be an N-end rule processing substrate as well. This may also be the cause of the increased import and assembly efficiency of B13 into the *Δoct1* mitochondria. A similar increase in assembly was previously observed when the complex I subunit carbonic anhydrase 2 (CA) was imported into mitochondria lacking this subunit (Li *et al.*, 2013). However, in the case of CA2, there was no CA2 within the mitochondria prior to import, whereas in the case of B13, mitochondria already contain some B13 subunits. Thus, the exact reason why the *Δoct1* mitochondria assemble B13 in a more efficient manner remains unclear. It could be due to a higher turnover and assembly of complex I in general to compensate for the instability of the B13 subunit. It could also be that OCT1 activity is quite slow under *in vitro* conditions, and so assembly is slower in the wild type as B13 must wait for OCT1 processing before being assembled into complex I. In *Δoct1* mitochondria lacking OCT1, assembly proceeds without OCT1 cleavage and appears more efficient. Determination of the exact role OCT1 plays in the import and assembly of B13 requires more work on how, exactly, the B13 subunit is inserted into the complex, and at what stage it is required for complex I assembly.

In previous work looking at the presequences of dual-targeted proteins, it has been suggested that they fall somewhere in between plastid transit peptides and mitochondrial presequences (Berglund *et al.*, 2009; Mitschke *et al.*, 2009; Ge *et al.*, 2014). However, most of this work has been performed on arbitrary lengths of ~70 amino acids, not using the actual presequences from known dual-targeted proteins. Thus, when we compared actual dual-targeted presequences to those of plant mitochondria, yeast mitochondria, and chloroplasts, they appear to more closely resemble chloroplast transit peptides in terms of presequence length and overall net charge. Interestingly, the majority of dual-targeted presequences have a consensus cleavage motif of RX(F/L)↓(S/A)(S/T/V/A), which more closely resembles that of mitochondrial presequences. This may reflect the fact that the majority of the dual-targeted presequences identified have come from isolated mitochondrial samples. Therefore, it is conceivable that analysing isolated chloroplasts in similar ways may identify different processing sites. Currently, there is little known about the processing of dual-targeted proteins in chloroplasts, but by looking at *in vitro* import studies of dual-targeted proteins into chloroplasts and mitochondria, it has been observed that the mature imported proteins are of similar sizes between organelles (Chew *et al.*, 2003; Duchene *et al.*, 2005; Berglund *et al.*, 2009). With regard to processing, the only dual-targeted protein to be analysed in any detail is the pea glutathione reductase (Rudhe *et al.*, 2004). In this study, it was concluded that SPP and MPP have different recognition determinants for the processing site (Rudhe *et al.*, 2004). However, this work has one major problem; the N-terminal sequences were not determined following MPP or SPP cleavage, and only SDS-gels were used for comparison (Rudhe *et al.*, 2004). Thus, it cannot be determined whether SPP or MPP generate the same N-terminus or not, and so it is still a possibility that dual-targeted proteins are processed at different sites between organelles. This is evidenced by the peptide deformylase and the Asp-tRNA synthetase, which have different N-termini in the different organelles. It is even possible that the dual-targeted proteins previously used in *in vitro* import assays possess different processing sites that are too close together (one or two amino acids) to be seen in SDS-PAGE gels. Thus, more work in this area is required to determine whether dual-targeted proteins have identical processing sites in both organelles or if they actually differ by one or two amino acids.

Overall, this study has demonstrated that *Arabidopsis* mitochondria contain both ICP55 and OCT1 intermediate peptidases. AtICP55 has an almost identical processing site to that of the Icp55 identified in yeast, and is probably responsible for a similar function. However, for AtOCT1, no consensus cleavage site was identified in the small number of substrates and the processing sites show little conservation with the yeast and mammalian Oct1 proteins. Therefore, more work is required to better characterize the activity of AtOCT1 in order to determine its exact physiological role. It was also demonstrated that the majority of dual-targeted proteins have processing sites similar to mitochondrial presequences.

## Supplementary material

Supplementary data can be found at *JXB* online.

Supplementary Table S1. Protein sequence alignments and subcellular predictions for AtICP55 and AtOCT1 proteins.

Supplementary Table S2. Mitochondrial N-terminal peptides identified from *Δicp55*, *Δicp1-1*, and *Δicp1-2* samples.

Supplementary Table S3. Mitochondrial proteins identified with no presequence.

Supplementary Table S4. Presequences from *Arabidopsis* mitochondria, chloroplasts, and dual-targeted proteins, and from yeast mitochondria. Peptides highlighted in green come from this study; all other peptides come from the published studies indicated in the text.

Supplementary Table 5. Comparison of N-terminal peptides from this and previous studies.

Supplementary Figure S1. Location of T-DNA insertions and RT-PCR analysis of knockouts. (A) The genomic organization of both AtICP55 and AtOCT1. Black boxes indicate exons, and the locations of T-DNA insertions are displayed. (B) To determine whether the T-DNA insertions were total knockouts, the full-length open reading frame of both AtICP55 and AtOCT1 was amplified from cDNA obtained from RNA isolated from the wild type (Col-0) and the respective T-DNA insertion lines.

Supplementary Data

## References

[CIT0001] Altschul SF Gish W Miller W Myers EW Lipman DJ 1990 Basic local alignment search tool. Journal of Molecular Biology 215, 403–410. 2231712 10.1016/S0022-2836(05)80360-2

[CIT0002] Bachmair A Finley D Varshavsky A 1986 In vivo half-life of a protein is a function of its amino-terminal residue. Science 234, 179–186. 3018930 10.1126/science.3018930

[CIT0003] Becker T Bottinger L Pfanner N 2012 Mitochondrial protein import: from transport pathways to an integrated network. Trends in Biochemical Sciences 37, 85–91. 22178138 10.1016/j.tibs.2011.11.004

[CIT0004] Berglund AK Pujol C Duchene AM Glaser E 2009 Defining the determinants for dual targeting of amino acyl-tRNA synthetases to mitochondria and chloroplasts. Journal of Molecular Biology 393, 803–814. 19733576 10.1016/j.jmb.2009.08.072

[CIT0005] Bienvenut WV Sumpton D Martinez A Lilla S Espagne C Meinnel T Giglione C 2012 Comparative large scale characterization of plant versus mammal proteins reveals similar and idiosyncratic N-alpha-acetylation features. Molecular and Cellular Proteomics 11, M111.015131 22223895 10.1074/mcp.M111.015131 PMC3433923

[CIT0006] Braun HP Binder S Brennicke A 2014 The life of plant mitochondrial complex I. Mitochondriondoi doi: 10.1016/j.mito.2014.02.006 10.1016/j.mito.2014.02.006 24561573

[CIT0007] Braun HP Emmermann M Kruft V Schmitz UK 1992 The general mitochondrial processing peptidase from potato is an integral part of cytochrome c reductase of the respiratory chain. The EMBO Journal 11, 3219–3227. 1324169 10.1002/j.1460-2075.1992.tb05399.x PMC556855

[CIT0008] Burkhart JM Schumbrutzki C Wortelkamp S Sickmann A Zahedi RP 2012 Systematic and quantitative comparison of digest efficiency and specificity reveals the impact of trypsin quality on MS-based proteomics. Journal of Proteomics 75, 1454–1462. 22166745 10.1016/j.jprot.2011.11.016

[CIT0009] Carrie C Giraud E Duncan O 2010 Conserved and novel functions for Arabidopsis thaliana MIA40 in assembly of proteins in mitochondria and peroxisomes. The Journal of Biological Chemistry 285, 36138–36148. 20829360 10.1074/jbc.M110.121202 PMC2975236

[CIT0010] Carrie C Small I 2013 A reevaluation of dual-targeting of proteins to mitochondria and chloroplasts. Biochimica et Biophysica Acta 1833, 253–259. 22683762 10.1016/j.bbamcr.2012.05.029

[CIT0011] Carrie C Whelan J 2013 Widespread dual targeting of proteins in land plants: when, where, how and why. Plant Signaling and Behavior doi: 10.4161/psb.25034 10.4161/psb.25034 PMC3999085 23733068

[CIT0012] Chacinska A Koehler CM Milenkovic D Lithgow T Pfanner N 2009 Importing mitochondrial proteins: machineries and mechanisms. Cell 138, 628–644. 19703392 10.1016/j.cell.2009.08.005 PMC4099469

[CIT0013] Chang W Soll J Bolter B 2014 A new member of the psToc159 family contributes to distinct protein targeting pathways in pea chloroplasts. Frontiers in Plant Science 5, 239. 24904628 10.3389/fpls.2014.00239 PMC4036074

[CIT0014] Chew O Whelan J Millar AH 2003 Molecular definition of the ascorbate-glutathione cycle in Arabidopsis mitochondria reveals dual targeting of antioxidant defenses in plants. The Journal of Biological Chemistry 278, 46869–46877. 12954611 10.1074/jbc.M307525200

[CIT0015] Colaert N Helsens K Martens L Vandekerckhove J Gevaert K 2009 Improved visualization of protein consensus sequences by iceLogo. Nature Methods 6, 786–787. 19876014 10.1038/nmeth1109-786

[CIT0016] Dessi P Rudhe C Glaser E 2000 Studies on the topology of the protein import channel in relation to the plant mitochondrial processing peptidase integrated into the cytochrome bc1 complex. The Plant Journal 24, 637–644. 11123802 10.1046/j.1365-313x.2000.00910.x

[CIT0017] Duchene AM Giritch A Hoffmann B Cognat V Lancelin D Peeters NM Zaepfel M Marechal-Drouard L Small ID 2005 Dual targeting is the rule for organellar aminoacyl-tRNA synthetases in Arabidopsis thaliana. Proceedings of the National Academy of Sciences, USA 102, 16484–16489. 10.1073/pnas.0504682102 PMC1283425 16251277

[CIT0018] Emanuelsson O Brunak S von Heijne G Nielsen H 2007 Locating proteins in the cell using TargetP, SignalP and related tools. Nature Protocols 2, 953–971. 17446895 10.1038/nprot.2007.131

[CIT0019] Gakh O Cavadini P Isaya G 2002 Mitochondrial processing peptidases. Biochimica et Biophysica Acta 1592, 63–77. 12191769 10.1016/s0167-4889(02)00265-3

[CIT0020] Gawryluk RM Chisholm KA Pinto DM Gray MW 2014 Compositional complexity of the mitochondrial proteome of a unicellular eukaryote (Acanthamoeba castellanii, supergroup Amoebozoa) rivals that of animals, fungi, and plants. Journal of Proteomics 109C, 400–416. 25026440 10.1016/j.jprot.2014.07.005

[CIT0021] Ge C Spanning E Glaser E Wieslander A 2014 Import determinants of organelle-specific and dual targeting peptides of mitochondria and chloroplasts in Arabidopsis thaliana. Molecular Plant 7, 121–136. 24214895 10.1093/mp/sst148

[CIT0022] Glaser E Dessi P 1999 Integration of the mitochondrial-processing peptidase into the cytochrome bc1 complex in plants. Journal of Bioenergetics and Biomembranes 31, 259–274. 10591532 10.1023/a:1005475930477

[CIT0023] Glaser E Sjoling S Tanudji M Whelan J 1998 Mitochondrial protein import in plants. Signals, sorting, targeting, processing and regulation. Plant Molecular Biology 38, 311–338. 9738973 10.1023/a:1006020208140

[CIT0024] Graciet E Wellmer F 2010 The plant N-end rule pathway: structure and functions. Trends in Plant Science 15, 447–453. 20627801 10.1016/j.tplants.2010.04.011

[CIT0025] Hawlitschek G Schneider H Schmidt B Tropschug M Hartl FU Neupert W 1988 Mitochondrial protein import: identification of processing peptidase and of PEP, a processing enhancing protein. Cell 53, 795–806. 2967109 10.1016/0092-8674(88)90096-7

[CIT0026] Hirst J Carroll J Fearnley IM Shannon RJ Walker JE 2003 The nuclear encoded subunits of complex I from bovine heart mitochondria. Biochimica et Biophysica Acta 1604, 135–150. 12837546 10.1016/s0005-2728(03)00059-8

[CIT0027] Hooper CM Tanz SK Castleden IR Vacher MA Small ID Millar AH 2014 SUBAcon: a consensus algorithm for unifying the subcellular localization data of the Arabidopsis proteome. Bioinformatics 30, 3356–3364. 25150248 10.1093/bioinformatics/btu550

[CIT0028] Huang S Taylor NL Whelan J Millar AH 2009 Refining the definition of plant mitochondrial presequences through analysis of sorting signals, N-terminal modifications, and cleavage motifs. Plant Physiology 150, 1272–1285. 19474214 10.1104/pp.109.137885 PMC2705053

[CIT0029] Isaya G Kalousek F Fenton WA Rosenberg LE 1991 Cleavage of precursors by the mitochondrial processing peptidase requires a compatible mature protein or an intermediate octapeptide. The Journal of Cell Biology 113, 65–76. 1672532 10.1083/jcb.113.1.65 PMC2288917

[CIT0030] Isaya G Miklos D Rollins RA 1994 MIP1, a new yeast gene homologous to the rat mitochondrial intermediate peptidase gene, is required for oxidative metabolism in Saccharomyces cerevisiae. Molecular Cell Biology 14, 5603–5616. 10.1128/mcb.14.8.5603-5616.1994 PMC359079 8035833

[CIT0031] Karimi M Inze D Depicker A 2002 GATEWAY vectors for Agrobacterium-mediated plant transformation. Trends in Plant Science 7, 193–195. 11992820 10.1016/s1360-1385(02)02251-3

[CIT0032] Kleffmann T Russenberger D von Zychlinski A Christopher W Sjolander K Gruissem W Baginsky S 2004 The Arabidopsis thaliana chloroplast proteome reveals pathway abundance and novel protein functions. Current Biology 14, 354–362. 15028209 10.1016/j.cub.2004.02.039

[CIT0033] Koop HU Steinmuller K Wagner H Rossler C Eibl C Sacher L 1996 Integration of foreign sequences into the tobacco plastome via polyethylene glycol-mediated protoplast transformation. Planta 199, 193–201. 8680308 10.1007/BF00196559

[CIT0034] Kruft V Eubel H Jansch L Werhahn W Braun HP 2001 Proteomic approach to identify novel mitochondrial proteins in Arabidopsis. Plant Physiology 127, 1694–1710. 11743114 PMC133574

[CIT0035] Kwasniak M Pogorzelec L Migdal I Smakowska E Janska H 2012 Proteolytic system of plant mitochondria. Physiologia Plantarum 145, 187–195. 22085399 10.1111/j.1399-3054.2011.01542.x

[CIT0036] Li B Takahashi D Kawamura Y Uemura M 2012 *a* . Comparison of plasma membrane proteomic changes of Arabidopsis suspension-cultured cells (T87 Line) after cold and ABA treatment in association with freezing tolerance development. Plant and Cell Physiology 53, 543–554. 22318864 10.1093/pcp/pcs010

[CIT0037] Li L Carrie C Nelson C Whelan J Millar AH 2012 *b* . Accumulation of newly synthesized F1 in vivo in arabidopsis mitochondria provides evidence for modular assembly of the plant F1Fo ATP synthase. The Journal of Biological Chemistry 287, 25749–25757. 22674576 10.1074/jbc.M112.373506 PMC3591127

[CIT0038] Li L Nelson CJ Carrie C Gawryluk RM Solheim C Gray MW Whelan J Millar AH 2013 Subcomplexes of ancestral respiratory complex I subunits rapidly turn over in vivo as productive assembly intermediates in Arabidopsis. The Journal of Biological Chemistry 288, 5707–5717. 23271729 10.1074/jbc.M112.432070 PMC3581425

[CIT0039] Lister R Carrie C Duncan O Ho LH Howell KA Murcha MW Whelan J 2007 Functional definition of outer membrane proteins involved in preprotein import into mitochondria. The Plant Cell 19, 3739–3759. 17981999 10.1105/tpc.107.050534 PMC2174869

[CIT0040] Millar AH Heazlewood JL Kristensen BK Braun HP Moller IM 2005 The plant mitochondrial proteome. Trends in Plant Science 10, 36–43. 15642522 10.1016/j.tplants.2004.12.002

[CIT0041] Mitschke J Fuss J Blum T Hoglund A Reski R Kohlbacher O Rensing SA 2009 Prediction of dual protein targeting to plant organelles. New Phytologist 183, 224–235. 19368670 10.1111/j.1469-8137.2009.02832.x

[CIT0042] Mossmann D Meisinger C Vogtle FN 2012 Processing of mitochondrial presequences. Biochimica et Biophysica Acta 1819, 1098–1106. 22172993 10.1016/j.bbagrm.2011.11.007

[CIT0043] Murcha MW Lister R Ho AY Whelan J 2003 Identification, expression, and import of components 17 and 23 of the inner mitochondrial membrane translocase from Arabidopsis. Plant Physiology 131, 1737–1747. 12692332 10.1104/pp.102.016808 PMC166929

[CIT0044] Naamati A Regev-Rudzki N Galperin S Lill R Pines O 2009 Dual targeting of Nfs1 and discovery of its novel processing enzyme, Icp55. The Journal of Biological Chemistry 284, 30200–30208. 19720832 10.1074/jbc.M109.034694 PMC2781575

[CIT0045] Nett JH Trumpower BL 1999 Intermediate length Rieske iron-sulfur protein is present and functionally active in the cytochrome bc1 complex of Saccharomyces cerevisiae. The Journal of Biological Chemistry 274, 9253–9257. 10092599 10.1074/jbc.274.14.9253

[CIT0046] Ou WJ Ito A Okazaki H Omura T 1989 Purification and characterization of a processing protease from rat liver mitochondria. The EMBO Journal 8, 2605–2612. 2684653 10.1002/j.1460-2075.1989.tb08400.x PMC401266

[CIT0047] Peltier JB Ytterberg AJ Sun Q van Wijk KJ 2004 New functions of the thylakoid membrane proteome of Arabidopsis thaliana revealed by a simple, fast, and versatile fractionation strategy. The Journal of Biological Chemistry 279, 49367–49383. 15322131 10.1074/jbc.M406763200

[CIT0048] Rudhe C Clifton R Chew O Zemam K Richter S Lamppa G Whelan J Glaser E 2004 Processing of the dual targeted precursor protein of glutathione reductase in mitochondria and chloroplasts. Journal of Molecular Biology 343, 639–647. 15465051 10.1016/j.jmb.2004.08.053

[CIT0049] Schweiger R Muller NC Schmitt MJ Soll J Schwenkert S 2012 AtTPR7 is a chaperone-docking protein of the Sec translocon in Arabidopsis. Journal of Cell Science 125, 5196–5207. 22899711 10.1242/jcs.111054

[CIT0050] Stames EM O’Toole JF 2013 Mitochondrial aminopeptidase deletion increases chronological lifespan and oxidative stress resistance while decreasing respiratory metabolism in S. cerevisiae. PLoS ONE 8, e77234. 24116217 10.1371/journal.pone.0077234 PMC3792884

[CIT0051] Tanz SK Castleden I Hooper CM Vacher M Small I Millar HA 2013 SUBA3: a database for integrating experimentation and prediction to define the SUBcellular location of proteins in Arabidopsis. Nucleic Acids Research 41, D1185–1191. 23180787 10.1093/nar/gks1151 PMC3531127

[CIT0052] Teixeira PF Glaser E 2013 Processing peptidases in mitochondria and chloroplasts. Biochimica et Biophysica Acta 1833, 360–370. 22495024 10.1016/j.bbamcr.2012.03.012

[CIT0053] Thingholm TE Palmisano G Kjeldsen F Larsen MR 2010 Undesirable charge-enhancement of isobaric tagged phosphopeptides leads to reduced identification efficiency. Journal of Proteome Research 9, 4045–4052. 20515019 10.1021/pr100230q

[CIT0054] Varshavsky A 2011 The N-end rule pathway and regulation by proteolysis. Protein Science 20, 1298–1345. 21633985 10.1002/pro.666 PMC3189519

[CIT0055] Venne AS Vogtle FN Meisinger C Sickmann A Zahedi RP 2013 Novel highly sensitive, specific, and straightforward strategy for comprehensive N-terminal proteomics reveals unknown substrates of the mitochondrial peptidase Icp55. Journal of Proteome Research 12, 3823–3830. 23964590 10.1021/pr400435d

[CIT0056] Vogtle FN Prinz C Kellermann J Lottspeich F Pfanner N Meisinger C 2011 Mitochondrial protein turnover: role of the precursor intermediate peptidase Oct1 in protein stabilization. Molecular Biology of the Cell 22, 2135–2143. 21525245 10.1091/mbc.E11-02-0169 PMC3128517

[CIT0057] Vogtle FN Wortelkamp S Zahedi RP 2009 Global analysis of the mitochondrial N-proteome identifies a processing peptidase critical for protein stability. Cell 139, 428–439. 19837041 10.1016/j.cell.2009.07.045

[CIT0058] von Heijne G 1986 Mitochondrial targeting sequences may form amphiphilic helices. The EMBO Journal 5, 1335–1342. 3015599 10.1002/j.1460-2075.1986.tb04364.x PMC1166945

[CIT0059] von Heijne G Steppuhn J Herrmann RG 1989 Domain structure of mitochondrial and chloroplast targeting peptides. European Journal of Biochemistry 180, 535–545. 2653818 10.1111/j.1432-1033.1989.tb14679.x

[CIT0060] Whelan J Hugosson M Glaser E Day DA 1995a Studies on the import and processing of the alternative oxidase precursor by isolated soybean mitochondria. Plant Molecular Biology 27, 769–778. 7727753 10.1007/BF00020229

[CIT0061] Whelan J Smith MK Meijer M Yu JW Badger MR Price GD Day DA 1995b Cloning of an additional cDNA for the alternative oxidase in tobacco. Plant Physiology 107, 1469–1470. 7770539 10.1104/pp.107.4.1469 PMC157290

[CIT0062] Wilce MC Bond CS Dixon NE Freeman HC Guss JM Lilley PE Wilce JA 1998 Structure and mechanism of a proline-specific aminopeptidase from Escherichia coli. Proceedings of the National Academy of Sciences, USA 95, 3472–3477. 10.1073/pnas.95.7.3472 PMC19860 9520390

[CIT0063] Yoon HS Hackett JD Ciniglia C Pinto G Bhattacharya D 2004 A molecular timeline for the origin of photosynthetic eukaryotes. Molecular Biology and Evolution 21, 809–818. 14963099 10.1093/molbev/msh075

[CIT0064] Zhang XP Sjoling S Tanudji M Somogyi L Andreu D Eriksson LE Graslund A Whelan J Glaser E 2001 Mutagenesis and computer modelling approach to study determinants for recognition of signal peptides by the mitochondrial processing peptidase. The Plant Journal 27, 427–438. 11576427 10.1046/j.1365-313x.2001.01108.x

[CIT0065] Zybailov B Rutschow H Friso G Rudella A Emanuelsson O Sun Q van Wijk KJ 2008 Sorting signals, N-terminal modifications and abundance of the chloroplast proteome. PLoS ONE 3, e1994. 18431481 10.1371/journal.pone.0001994 PMC2291561

