# Supplementary material for: Identification of cleavage sites and substrate proteins for two mitochondrial intermediate peptidases in Arabidopsis thaliana
Source: J Exp Bot. 2015 Mar 1;66(9):2691–708. doi: 10.1093/jxb/erv064 (PMC4986872; doi:10.1093/jxb/erv064)
Supplement: Supplementary Data [file supp_66_9_2691__index.html]

Identification of cleavage sites and substrate proteins for two mitochondrial intermediate peptidases in Arabidopsis thaliana — Identification of cleavage sites and substrate proteins for two mitochondrial intermediate peptidases in Arabidopsis thaliana — Supplementary Data 

# Identification of cleavage sites and substrate proteins for two mitochondrial intermediate peptidases in *Arabidopsis thaliana*

## Supplementary Data

Data files

**Files in this Data Supplement:**

- Supplementary Data - Supplementary Data
- Supplementary Data - Supplementary Data
- Supplementary Data - Supplementary Data
- Supplementary Data - Supplementary Data
- Supplementary Data - Supplementary Data
- Supplementary Data - Supplementary Data
