# Supplementary figures and images for: Identification of cleavage sites and substrate proteins for two mitochondrial intermediate peptidases in Arabidopsis thaliana
Source: J Exp Bot. 2015 Mar 1;66(9):2691–708. doi: 10.1093/jxb/erv064 (PMC4986872; doi:10.1093/jxb/erv064)

A)

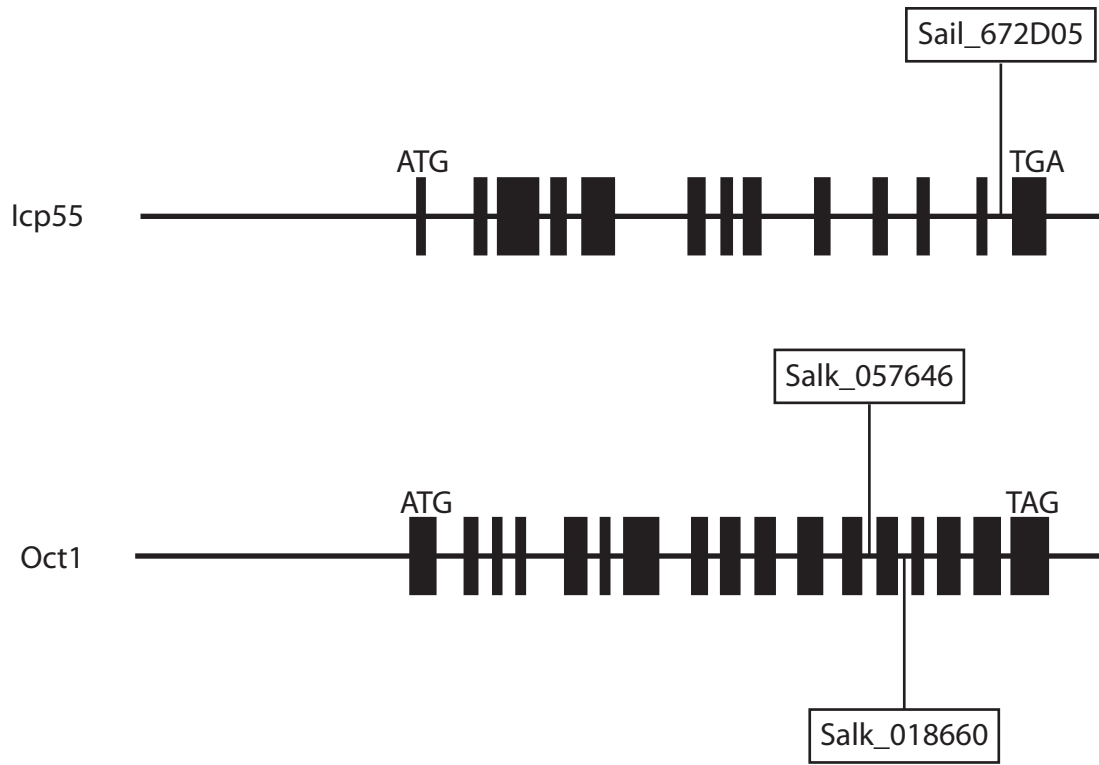

B)

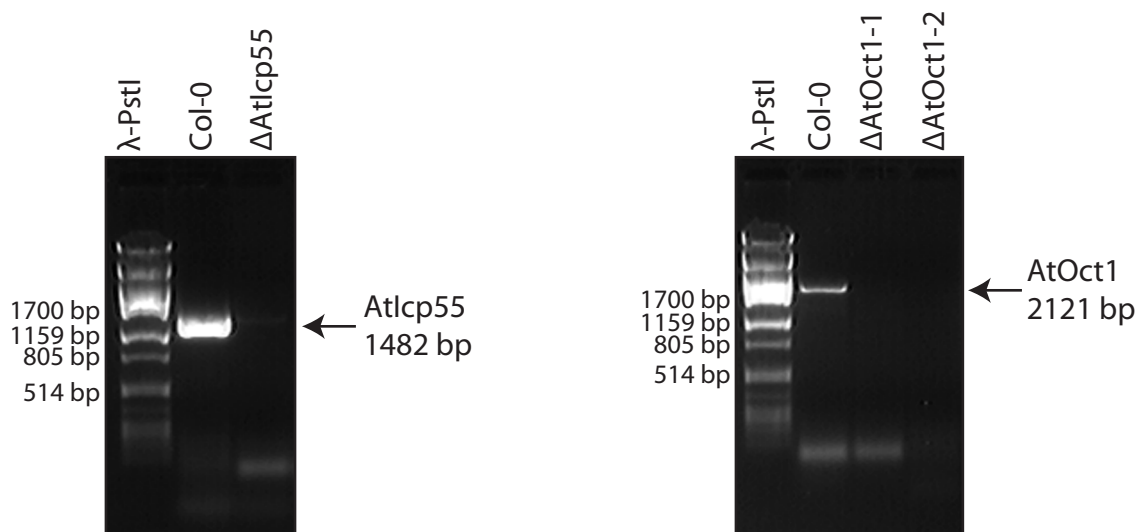

Supplementary Figure 1

Supplement: Supplementary Data [file supp_erv064_jexbot142661_file001.pdf]
